# Continuous biomarker monitoring with single molecule resolution by measuring free particle motion

**DOI:** 10.1038/s41467-022-33487-3

**Published:** 2022-10-13

**Authors:** Alissa D. Buskermolen, Yu-Ting Lin, Laura van Smeden, Rik B. van Haaften, Junhong Yan, Khulan Sergelen, Arthur M. de Jong, Menno W. J. Prins

**Affiliations:** 1 Department of Biomedical Engineering, Eindhoven University of Technology, Eindhoven, the Netherlands; 2 Institute for Complex Molecular Systems (ICMS), Eindhoven University of Technology, Eindhoven, the Netherlands; 3 Department of Applied Physics, Eindhoven University of Technology, Eindhoven, the Netherlands; 4 Helia Biomonitoring, Eindhoven, the Netherlands

**Keywords:** Sensors and probes, Sensors, Biosensors, Biomarkers

## Abstract

There is a need for sensing technologies that can continuously monitor concentration levels of critical biomolecules in applications such as patient care, fundamental biological research, biotechnology and food industry, as well as the environment. However, it is fundamentally difficult to develop measurement technologies that are not only sensitive and specific, but also allow monitoring over a broad concentration range and over long timespans. Here we describe a continuous biomolecular sensing methodology based on the free diffusion of biofunctionalized particles hovering over a sensor surface. The method records digital events due to single-molecule interactions and enables biomarker monitoring at picomolar to micromolar concentrations without consuming any reagents. We demonstrate the affinity-based sensing methodology for DNA-based sandwich and competition assays, and for an antibody-based cortisol assay. Additionally, the sensor can be dried, facilitating storage over weeks while maintaining its sensitivity. We foresee that this will enable the development of continuous monitoring sensors for applications in fundamental research, for studies on organs on a chip, for the monitoring of patients in critical care, and for the monitoring of industrial processes and bioreactors as well as ecological systems.

## Abstract (web summary)

Various applications would benefit from the ability to continuously measure biomolecules. Here the authors describe a biosensing technology based on the free diffusion of biofunctionalized particles hovering over a sensor surface, which enables monitoring of analytes (pM-µM) over long timespans.

## Introduction

The﻿ ability to continuously monitor concentration levels of key molecules in dynamic biological systems would offer exciting opportunities in applications such as fundamental biological research^1–5^, studies on organs-on-a-chip^6,7^, monitoring of patients in critical care^8–12^, and monitoring of industrial processes^13,14^ and bioreactors^15,16^ as well as ecological systems^17–19^. Continuous monitoring sensors are commercially available for glucose, but glucose is present at high concentrations (millimolar)^20^. It is fundamentally difficult to develop bioanalytical technologies that can continuously measure molecules at low concentrations (micro-, nano-, picomolar) over long timespans. Enzyme-linked immunosorbent assays (ELISA) are well-established and sensitive, but in every assay reagents are consumed, such as antibodies and enzymes. The repeated use of reagents complicates applications that require frequent measurements over long periods. On the other hand, some technologies can operate continuously without consuming reagents, such as surface plasmon resonance^21^, redox cycling^22,23^ and quartz crystal microbalance^24^, but these methods have not been designed for monitoring biomolecules at very low concentrations.

Recently a technology for biomolecular monitoring was proposed based on particles coupled to a substrate (i.e. a sensor surface) via a dsDNA tether^25–28^. Target molecules bind reversibly to the particle and/or substrate, resulting in single-molecular bonds that are recorded via changes in the Brownian motion of the tethered particle. However, the molecular tether is an essential element in the sensor design, so breakage and detachment of tethers lead to a loss of sensor functionality. Furthermore, the presence of the tether restricts the rotational freedom of the particle with respect to the substrate, which can limit the sensitivity and precision of the sensor^29^.

Here, we demonstrate a biomolecular monitoring methodology that is based on free diffusion of particles near a substrate, which we refer to as free-diffusion-based biosensing by particle mobility (f-BPM). The proximity of particles to the substrate is not imposed by a molecular tether but by gravitational force, which facilitates free translational and rotational motion of the particles. The particles are tracked over long periods of time, reversible single-molecular interactions are continuously probed with high signal contrast, and furthermore the total surfaces of particles and sensing substrate are used.

In this paper we describe the basic concepts of the sensing methodology and demonstrate it for the continuous monitoring of macromolecules and small molecules, in sandwich and competition assay formats, and with biomarker concentrations across the picomolar, nanomolar and micromolar range. We demonstrate the versatility of the technology by comparing different particle sizes, data analysis methods, surface chemistries, affinity binders and sample matrices. In addition, we present a cartridge preparation method for long-term storage and immediate use of the sensors. Finally, we discuss the prospects of this sensing technology in clinical, industrial, and environmental monitoring applications.

## Results

### Sensing principle

**Fig. 1:**
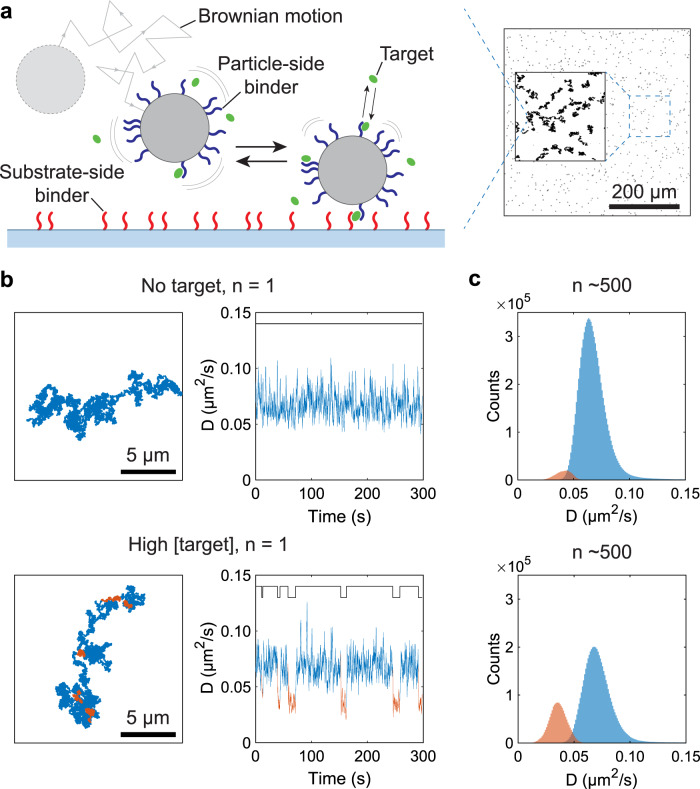
Basic principle of continuous biomarker monitoring based on measuring diffusional motion of biofunctionalized particles hovering over a substrate. The particles exhibit reversible target-induced molecular interactions with the substrate. **a** Microparticles (Dynabeads) are functionalized with particle-side binders (blue). The particles diffuse in the vicinity of a substrate functionalized with substrate-side binders (red). The binders (e.g. ssDNA or antibodies) have a specific affinity to target molecules (green; ranging from small molecules to macromolecules). Target-induced sandwich complexes are reversibly formed and cause the particles to switch between unbound and bound states. The particles exhibit free Brownian motion in the unbound state and confined Brownian motion in the bound state. The right panel shows a microscopy image of ~500 particles in the field of view (single frame). The inset shows the reconstructed in-plane trajectories of a random subset of particles (*n* = ~25) tracked for 300 s (1800 frames). In this experiment, the particles have a diameter of 2.8 μm. **b** Experimental data for a sandwich system with oligonucleotide binders and target. Left: Trajectories of single particles in absence (top) and presence (bottom) of target molecules in solution. The orange traces in the bottom panel indicate bound states caused by target-induced sandwich bonds. Right: Effective diffusivity *D* as a function of time based on the in-plane displacements derived from the particle trajectories. In the absence of analyte (top) the particles typically exhibit free Brownian motion. In the presence of analyte (bottom) particles show transitions from unbound (blue) to bound (orange) states and back. Attributed state transitions are indicated by binary step functions (black line at top). **c** Distributions of *D* of ~500 particles showing unbound state (blue) and bound state (orange) populations in absence (top) and presence (bottom) of target molecules in solution. Source data are provided as a Source Data file.

The molecular monitoring concept is based on continuous tracking of the Brownian motion of biofunctionalized particles hovering over a sensor surface. Figure 1 describes the basic principles for a sandwich assay configuration. Here, particles (Dynabeads) and the sensor surface are functionalized with affinity binders (ssDNA or antibodies) specific to the target molecule (ssDNA, cortisol). Particles are tracked over time using widefield video microscopy at ×10 magnification using a framerate of 60 Hz (Fig. 1a). Samples are typically incubated for two minutes, followed by recording particle motion in one to three blocks of five minutes. When target molecules are absent, the particles exhibit free Brownian motion with a corresponding diffusivity *D* (μm^2^/s). In the presence of target, affinity-based target-binder sandwich complexes are reversibly formed between the particle and the sensor surface, intermittently restricting the particle motion to a confined area with a significant decrease of the effective *D*. To distinguish the two characteristic motion types—free Brownian motion and confined Brownian motion—particle trajectories are reconstructed by determining particle centre positions in every recorded video frame. In post-processing, the effective *D* is determined as a function of time from the mean squared displacement (MSD) of the particles (see Supplementary Note 1.1). Periods with high *D* relate to free Brownian motion (>0.15 μm^2^/s for 1 μm particles, >0.05 μm^2^/s for 2.8 μm particles) and periods with low *D* to confined Brownian motion (bound state). Transitions between high and low *D* (Fig. 1b, Supplementary Fig. 3) are identified using Deep Learning analysis methods (explained in more detail in Supplementary Notes 2.2–2.4) and reflect the underlying single-molecule binding and unbinding events.

The switching activity, defined as the average number of binding and unbinding events per particle per unit of time, depends on the target concentration. In the case of a sandwich assay, the switching activity is low for low target concentration and increases with higher target concentrations (Fig. 1b). Typically, between 500 and 1000 particles are imaged and tracked simultaneously. Due to the large sample size, the distributions of *D* measured in the unbound states (blue) and the bound states (orange) show bell-shaped curves with a Gaussian-like distribution (Fig. 1c). The width of the curves represents the variability in *D* due to particles diffusing at various distances from the surface. The hydrodynamic interaction between the particle and the substrate causes the particle diffusivity *D* to be lower when a particle is closer to the substrate (Supplementary Fig. 1). In the absence of target most particles diffuse freely (blue curve in Fig. 1c, top), and with increasing target concentration the fraction with a low *D* increases due to the occurrence of bound states (orange curve in Fig. 1c, bottom).

### Influence of particle size

**Fig. 2:**
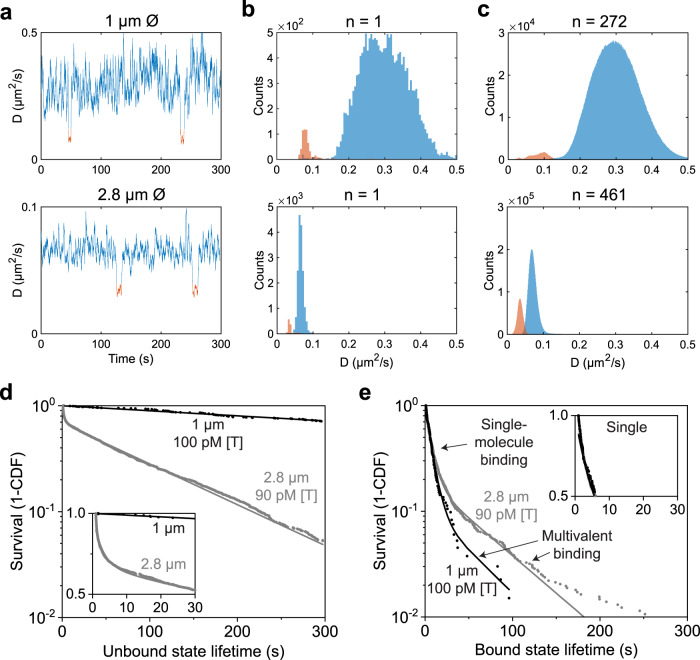
Diffusivity-time traces, histograms, and state lifetimes for an oligonucleotide sandwich assay using particles with a diameter of 1 and 2.8 μm. **a** Diffusivity *D* measured over a 5-min period showing unbound states (blue) and bound states (orange). **b** Distributions of *D* derived from the single-particle traces in panel **a**. The characteristic *D* of 1 and 2.8 μm particles differ by approximately a factor 4. **c** *D* distributions for hundreds of particles. **d** Distribution of unbound state lifetimes plotted as survival curves (1 - CDF, with CDF = Cumulative Distribution Function), for 1 and 2.8 μm particles with similar biofunctionalization and target concentration ([T] = [ssDNA target], Supplementary Table 2). Larger particles show shorter unbound state lifetimes than smaller particles for comparable conditions. The inset shows a zoom in of the data on linear scales. **e** Survival plot as in panel **d**, for bound state lifetimes. Curve segments are attributed to short-lived monovalent bonds and longer-lived multivalent bonds. The inset shows a zoom in of the data on linear scales, highlighting that the bound state lifetime of single-molecular bonds does not depend on the size of the particles. Source data are provided as a Source Data file.

The size of the particles is important for the sensing methodology because it influences their Brownian motion as well as the proximity to the substrate. Figure 2 shows the distributions of diffusive properties of commercially available particles with 1 μm (Dynabeads MyOne C1 Streptavidin) and 2.8 μm (Dynabeads M-270 Streptavidin) diameter for an oligonucleotide sandwich assay. For both particle sizes, the diffusivity-time traces (Fig. 2a) show clear transitions between unbound states (blue) and bound states (orange). The diffusivity of the 2.8 μm particles is lower and shows a narrower distribution compared to the 1 μm particles, caused by the differences in hydrodynamic interactions and proximity to the substrate (Supplementary Fig. 2). Panels b and  c of Fig. 2 show distributions of the effective diffusivity for single particles and for ensembles of hundreds of particles. Clearly different distributions of diffusivity are observed for unbound (blue) and bound (orange) states, and the distributions for single particles and ensembles of particles are similar, indicating that particle-to-particle variations play a minor role.

Significant changes in the diffusivity-time traces enable the identification of single-molecule binding and unbinding events, and measurements of unbound and bound state lifetimes (Supplementary Note 2). The unbound state lifetimes relate to the effective association rate of particles, which depends on the concentration of targets in solution, on the densities of binders on particle and substrate, and on the hit rate between particle and substrate. The hit rate is determined by the particle diffusivity and the average distance between particle and substrate. The average distance is dictated by the barometric height distribution, with a mean of ~60 nm for the 2.8 μm particles, and ~1 μm for the 1 μm particles (Supplementary Table 2 and Supplementary Note 3.1).

Larger particles are on average closer to the substrate, so higher effective association rates and therefore shorter unbound state lifetimes are expected. This is confirmed by the cumulative distribution function (CDF) plots of the observed lifetimes in Fig. 2d, which show a steeper curve and therefore a shorter mean unbound state lifetime for the 2.8 μm particles than for the 1 μm particles. Figure 2e shows survival curves of the bound state lifetimes for picomolar target concentrations. At such low target concentrations most particles are bound via a single interaction with the substrate (single-molecular bonds) and only a small fraction forms multivalent bonds with the substrate. The initial slopes and corresponding mean bound state lifetimes are independent of particle diameter, as expected for single-molecular bonds. The secondary segments, labelled multivalent binding, are explained in more detail in Supplementary Note 2.2.

### Oligonucleotide sandwich assay

**Fig. 3:**
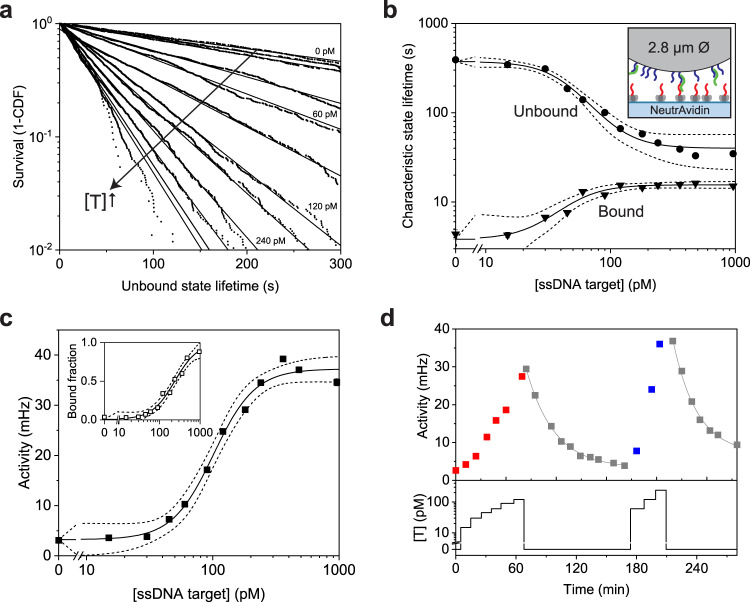
Oligonucleotide sandwich assay using 2.8 μm particles. **a** Survival curves of the characteristic unbound state lifetimes show a dependency on the target concentration [T]. With increasing ssDNA target concentration, the curves become steeper (black arrow), corresponding to shorter lifetimes. Data are fitted with a single exponential curve (see Supplementary Note 2.2) to extract the characteristic state lifetimes. **b** Characteristic unbound state lifetimes (circles) depend on the target concentration in a range of 15–300 pM. The sigmoidal fit (see “Methods” for fit equation) yields an EC50 of 45 ± 7 pM. The bound state lifetimes (triangles) depend weakly on target concentration. At low target concentrations, bound state lifetimes result mostly from short-lived, non-specific interactions. A gradual transition to specific bound states is observed. Data are presented as mean ± SE. The error bars are mostly smaller than the marker size. The inset shows the molecular design of the sensor, with NeutrAvidin on the substrate functionalized with partially single-stranded DNA binders (red) and with 2.8 μm particles functionalized with ssDNA binders (blue). The ssDNA target strand (green) can form sandwich complexes (via DNA hybridization) between the binders on particle and substrate (for details see Supplementary Note 3.2). **c** Measured activity as a function of target concentration, with a sigmoidal fit with an EC50 of 101 ± 7 pM. The inset shows the response expressed as the bound fraction, with an EC50 of 238 ± 39 pM. **d** Continuous monitoring of an applied concentration-time profile. The bottom panel shows the applied target concentration as a function of time; the top panel shows the measured switching activity. The relaxation curves are fitted with exponential decay functions (grey). The characteristic relaxation times are 28 ± 1 and 24 ± 2 min, and sensor functionality is retained after exposure to the blank solutions. Activity data in panels **c** and **d** are presented as mean ±  SEM. Error bars for the activity are generally smaller than the marker size. The dashed lines in panels **b** and **c** indicate the 95% confidence interval of the sigmoidal fits and determine the error in the reported EC50 values. Source data are provided as a Source Data file.

The sensitivity and reversibility of a sandwich-type oligonucleotide monitoring assay was further investigated with 2.8 μm particles (Supplementary Note 3.1). Particles and substrate were functionalized with ssDNA-biotin strands as binders, such that ssDNA targets can form sandwich complexes (via DNA hybridization) between the ssDNA binders on particle and substrate (inset Fig. 3b and Supplementary Note 3.2).

With increasing target concentrations, the slopes of the unbound state lifetime curves become steeper (Fig. 3a), indicating a decreasing mean lifetime and thus a higher rate of sandwich complex formation. The characteristic unbound state lifetimes depend on the target concentration in a range of 15–300 pM, with an EC50 of 45 ± 7 pM (Fig. 3b). The characteristic bound state lifetimes (triangles) also show a dependence on target concentration. At low target concentrations, the bound states originate from short-lived non-specific interactions. Above ~70 pM the bound state lifetime stabilizes, caused by target-induced specific bonds between particles and substrate (Fig. 3b).

Figure 3c shows how the switching activity, i.e., the average number of binding and unbinding events per particle per unit of time, depends on the target concentration. The graph shows sensitivity in the range of 30–500 pM with an EC50 of 101 ± 7 pM. The inset of Fig. 3c shows the fraction of bound particles against the target concentration, with an EC50 value of 238 ± 39 pM. Comparing both dose-response curves, a measurement of activity is most sensitive while a measurement of bound fraction gives a wider dynamic range. The dynamic range of the sensor can be tuned over several orders of magnitude by increasing or decreasing the binder areal density; in this case of the substrate-side binders (Supplementary Fig. 11). The specificity of the sensor was tested by spiking a high concentration of a mismatched ssDNA target (20-nt with 3 nucleotides difference), which showed no particle binding (Supplementary Fig. 10).

Continuous monitoring of various target concentrations as well as sensor reversibility are demonstrated in Fig. 3d. The activity increases with increasing target concentration (15–120 pM) and returns to the baseline when target is removed. The sensor is reversible within 90 min, with a characteristic relaxation time of 28 ± 1 min (single exponential decay fit, grey line). The target response and return to baseline were repeated a second time (up to 240 pM target), demonstrating the possibility to repeatedly monitor varying target concentrations over long timespans. Similar responses are observed in the bound fraction and unbound state lifetimes (Supplementary Fig. 8), demonstrating the consistency of sensor response using multiple readout parameters.

### Oligonucleotide competition assay

**Fig. 4:**
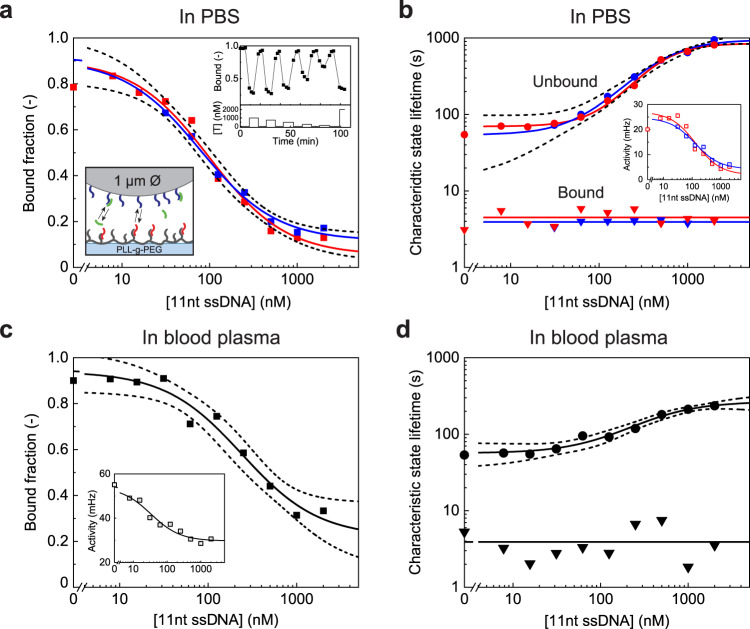
Oligonucleotide competition assay in buffer and in filtered blood plasma, using 1 μm particles. **a** The bound fraction as a function of target concentration ([T] = [11-nt ssDNA]). The red and blue squares represent two dose-response curves measured consecutively, demonstrating the reversibility of the sensor. The EC50 of the two dose-response curves fitted with sigmoidal curves (see “Methods” for fit equation) are 79 ± 22 nM and 100 ± 33 nM, respectively. The top inset shows the time-dependent response of bound fraction to alternating high and low analyte concentrations, demonstrating the fast response time of the sensor. The bottom inset shows the molecular design of the assay, with partially single-stranded DNA particle-side binders (blue), partially single-stranded DNA substrate-side binders (red), and ssDNA target molecules (green). The details of the surface chemistry and DNA hybridization are included in Supplementary Note 3.2. **b** Characteristic unbound state lifetimes are dependent on the target concentration in a range of 10 to 2000 nM. The unbound state lifetime curve has a sigmoidal shape with an EC50 of 461 ± 41 nM. The bound state lifetimes (triangles) are independent of the target concentration, with an average of 4 ± 1 s (solid horizontal lines). The inset shows the dose-response curves plotted as switching activity; the fitted EC50 values are 110 ± 78 nM and 127 ± 58 nM. **c** Bound fraction measured in 50 kDa spin-filtered bovine blood plasma, with an EC50 of 241 ± 83 nM. The inset shows the switching activity as a function of target concentration; the fitted EC50 value is 33 ± 15 nM. **d** Characteristic unbound and bound state lifetimes measured in filtered bovine blood plasma. Fitting the characteristic unbound state lifetime with a sigmoidal curve gives an EC50 of 421 ± 130  nM. The characteristic bound state lifetime is independent of target concentration with an average of 4 ± 2 s. Activity data in panels **b** and **c** are presented as mean ± SEM. Error bars for the activity are generally smaller than the marker size. The dashed lines in panels **b** and **c** indicate the 95% confidence interval of the sigmoidal fits and determine the error in the reported EC50 values. Source data are provided as a Source Data file.

Sandwich assays are suitable for measuring analyte molecules that are large enough to have two spatially separate binding sites. The f-BPM technique can also be used for measuring small molecules with only one binding site, by implementing a competition assay format. Figure 4 shows a competition assay for measuring small ssDNA fragments. The molecular design of the sensor is sketched in the bottom inset of Fig. 4a and Supplementary Figs. 6 and 7. The DBCO-tagged ssDNA substrate-side binders (red) are coupled to a PLL-g-PEG layer (grey) via the integrated azide groups using click chemistry^27^. The ssDNA particle-side binders (blue) are hybridized to a ssDNA-biotin linker coupled to 1 μm particles via biotin-streptavidin bonds. The substrate-side and particle-side binders exhibit reversible 9 bp hybridization, causing the particles to switch rapidly between unbound and bound states. Note that the particle-side binders are applied with a very low density (about 1000 times lower than in the ssDNA sandwich assay) to avoid irreversible multivalent bonds between particle and substrate. Hence, at zero analyte concentration, the particles show a high switching activity. Yet in the presence of the 11-nt analyte (green), the binding region on particle-side binders is blocked, causing a decrease in the particle switching activity.

Figure 4 shows the sensor response in buffer and in filtered blood plasma. Since the analytes (green) compete with the analogues (red) for binding to the particle-side binders (blue), the sensor shows an inverted response as compared to the sandwich assay: higher analyte concentration leads to lower switching activity. Panels a and b of Fig. 4 show how the three main readout parameters (bound fraction, unbound state lifetime, and switching activity) depend on the analyte concentration. Two profiles of gradually increasing target concentration were measured consecutively (red and blue data; Supplementary Fig. 9). The red and blue curves are very similar, showing that the sensor is reversible and that the molecular functionality and sensor response are consistent over time. The top inset in Fig. 4a shows the response of the sensor to alternating high and low analyte concentrations, demonstrating the fast response time of the sensor.

The characteristic bound state lifetimes (Fig. 4b, triangles) do not depend on the analyte concentration, indicating that the bound states are caused by one and the same single-molecular interaction. The curve of the unbound state lifetimes has a sigmoidal shape. At low target concentrations, the rate of particle-substrate binding is governed by the density of binder molecules, giving the plateau of a mean unbound state lifetime of ~70 s. At higher concentrations, the targets occupy part of the binder molecules causing the mean unbound state lifetime to increase. At concentrations above 1 μM, the unbound state lifetimes stabilize at ~850 s, caused by the finite duration of the measurement time.

To study the capability of detecting analytes in biologically relevant media, we performed the competition assay also in filtered undiluted blood plasma (Fig. 4c, d). The EC50 values measured in plasma and in buffer show good correspondence, indicating that the specific molecular interactions behave similarly in the two matrices. A difference is that there are more non-specific interactions in blood plasma, causing a slightly higher bound fraction and switching activity. The latter leads to lower characteristic unbound state lifetimes which are mostly observed at high analyte concentrations where non-specific interactions of the particles become more important with respect to specific interactions.

### Cortisol competition immunoassay

**Fig. 5:**
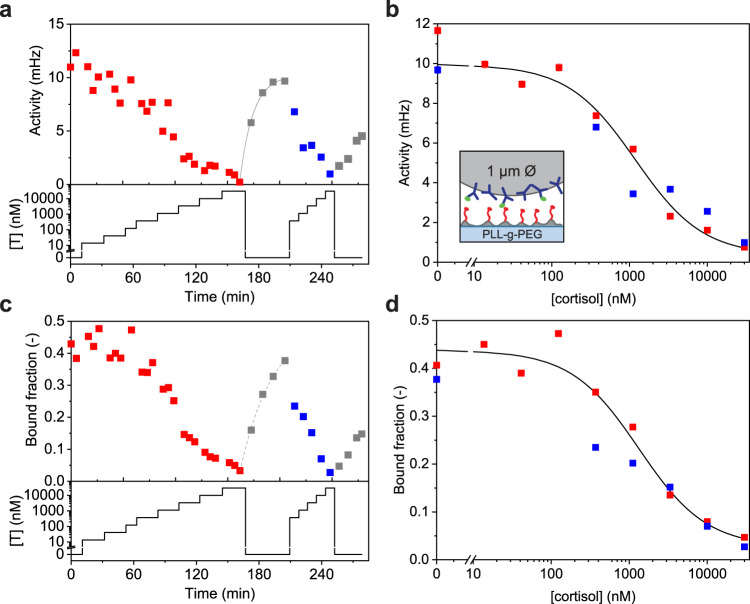
Cortisol competition immunoassay. **a** Dynamic sensor response to changes of cortisol concentration [T] including reversibility. Bottom panel: cortisol concentrations were applied over time in a stepwise increasing fashion, followed by blank solutions and a second cortisol concentration series. Top panel: the switching activity measured as a function of time. The grey line represents a single-exponential fit with a characteristic relaxation time of 13 ± 1 min. **b** Dose-response curve of cortisol measured in buffer, obtained from the same data as shown in panel **a**. A sigmoidal curve was fitted with an EC50 of 1.2 ± 0.5 μM (black line). The inset shows a schematic representation of the assay. Biotinylated antibodies (blue) coupled to 1 μm particles via biotin-streptavidin bonds, and cortisol-ssDNA conjugates (analogues, red) hybridized to DNA which is covalently coupled to a PLL-g-PEG layer using click chemistry (DBCO-azide). The reversible binding of antibodies to the analogues results in transient binding of particles to the substrate. In the presence of free cortisol (green), the antibodies bind to cortisol instead of the analogue, causing less binding of the particles to the substrate. **c** Same experiment as in panel **a**, but here the bound fraction is reported on the y-axis. Dashed grey line indicates sensor relaxation. **d** Dose-response curve obtained from the data shown in panel **c**. A sigmoidal fit is shown (black line) with an EC50 of 1.3 ± 0.2 μM. Activity data in panels **a** and **b** are presented as mean ± SEM. Error bars for the activity are generally smaller than the marker size. Source data are provided as a Source Data file.

Cortisol is a steroid hormone that is used as a clinical biomarker for stress and inflammation. Cortisol levels are normally measured in centralized laboratories using antibody-based ELISA assays. Here we demonstrate that cortisol can be measured continuously in an f-BPM assay using commercially available antibodies. Particles with 1 μm diameter were coated with cortisol-binding antibodies as particle-side binders and the substrate was coated with cortisol analogues (see inset Fig. 5; Supplementary Figs. 6, 7 and 12).

Figure 5 shows cortisol monitoring data over a period of 4.5 h. The cortisol concentration was varied between 0 and 30 μM and the switching activity (panels 5a, b) and bound fraction (panels 5c, d) were recorded. Both signals decrease with increasing analyte concentrations and increase with decreasing analyte concentrations, in agreement with competition assay functionality. The activity and bound fraction curves behave consistently, but the bound fraction shows more variability at low cortisol concentrations, potentially due to contributions of non-specific interactions. Between the two measurement series with stepwise increasing cortisol concentrations (red, blue), buffer solutions without cortisol were applied with 5- to 10-min intervals (grey data points). Here, the activity data show an exponential relaxation behaviour with a characteristic relaxation time of 13 ± 1 min (grey line). The dose-response curves (Fig. 5b, d) show that the sensor responds to cortisol concentrations in the range of 300 nM to 30 μM with an EC50 of 1.2 ± 0.5 μM (activity) and 1.5 ± 0.6 μM (bound fraction). The demonstrated sensitivity range and relaxation time are relevant for applications such as monitoring of stress, hormonal diseases, immune response, and peri-operative patient care^30–35^.

### Sensor conservation for long-term storage

**Fig. 6:**
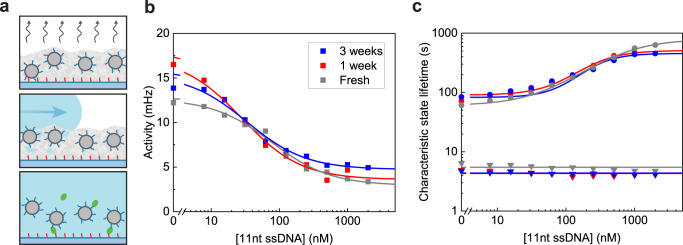
Sensor design with conservation layer. **a** Schematic of the processes to dry and rehydrate the particles. The grey layer represents the disaccharide layer (sucrose and trehalose) with 1 μm particles functionalized with particle-side binders (blue), deposited on a PLL-g-PEG surface with substrate-side binders (red). In the top panel, the disaccharide layer with particles is stored in a vacuum chamber and gradually dehydrated for long-term storage. In the middle panel, the disaccharide layer is rehydrated and the sensor chamber is washed with buffer. In the bottom panel, sample with target molecules is added to the sensor chamber and measurements are performed. **b** Sensor response characteristics for different storage conditions. The sensors stored for 1 and 3 weeks (red and blue data respectively) had a disaccharide layer. The control sample (fresh, grey data) was prepared without any sugar layer. The EC50 values of the three dose-response curves (fresh, one week, three weeks) are 77 ± 15 nM, 29 ± 5 nM and 30 ± 6 nM respectively. Data are presented as mean ± SEM. Error bars are generally smaller than the marker size. **c** Characteristic unbound state lifetimes are dependent on the target concentration in a range of 4 nM to 2 μM. The characteristic bound state lifetimes do not depend on concentration, with an average of 5 ± 1, 4 ± 1, and 4 ± 1 s (fresh, one week, three weeks). Source data are provided as a Source Data file.

The shelf life of biosensors depends on the retention of the functionality of the molecular components used. This may vary, depending on the coupling chemistry, the storage conditions and the intrinsic stability of the biomolecules. One way to preserve biomolecules is by embedding them in a sugar matrix. We investigated various sugar mixtures (sucrose/trehalose, glucose/fructose and pullulan/trehalose) according to the procedure sketched in Fig. 6a. The sucrose/trehalose mixture rehydrates easily, gave few stuck particles and the best uniformity. With a vacuum drying procedure, the glassy layer containing sucrose/trehalose has a homogeneous thickness throughout the cartridge. When the sucrose/trehalose layer rehydrates, about 90% of the particles regain their mobility and their biosensing functionality.

Panels b and c of Fig. 6 show dose-response curves measured with a freshly prepared sensor (grey) and with sensors that were stored in a sugar-matrix for one or three weeks (red and blue, each measured in duplicate). The sensors exhibit similar dose-response curves in terms of switching activity and state lifetimes, indicating that the sensors perform reproducibly, and that the drying and rehydration processes do not significantly affect the sensor performance. The results show that the sensor is suited for long-term storage in sugar matrices and for immediate use upon rehydration.

## Discussion

We described a continuous biomolecular sensing methodology based on the free diffusion of biofunctionalized particles hovering over a sensor surface. Digital events due to single-molecule interactions are recorded with high contrast using Deep Learning analysis methods, enabling biomarker monitoring at picomolar to micromolar concentrations. We studied sensor performance for particles of various sizes (∅ 1 and 2.8 μm), because the size affects the particle diffusion constant, the height distribution above the sensor surface, and the capacity to carry binder molecules. The sensing methodology was demonstrated for DNA-based sandwich and competition assays, for an antibody-based cortisol assay, and for a dry-stored sensor.

The BPM technology addresses the fundamental challenge to develop measurement methods that are sensitive, specific, and allow for the continuous monitoring of biomolecular concentrations over long timespans. The biomolecular interactions are reversible and therefore the measurement methodology can operate without consuming any reagents and without applying washing steps between different samples. Benchmark methodologies such as classical ELISAs^36^ and digital ELISAs^37,38^ can be very sensitive, but with every assay these methods consume biochemical reagents such as enzymes, antibodies and particles. Methods that measure optical refractive index (such as surface plasmon resonance, SPR)^21^ or acoustic parameters (such as quartz crystal microbalance, QCM)^24^ allow for continuous signal generation, but when assays are used without reagents and without washing steps, the sensitivities that can be achieved are limited. To achieve high sensitivities, typically signal amplification steps are applied, involving (bio)chemical reagents that are added in every assay^21^. Electrical biosensing methods based on redox cycling (such as electrochemical aptamer-based sensing, E-AB) use conformational changes of binder molecules to continuously measure analyte molecules. Monitoring of micromolar and nanomolar concentrations has been shown^22,39–41^, but the continuous monitoring of picomolar concentrations has not been demonstrated. BPM is suited for measuring large as well as small molecules, is sensitive in the micromolar, nanomolar and picomolar range while maintaining reversibility, and does not consume any reagents during sensor use. Furthermore, it allows for detection in filtered plasma; more complex matrices will be studied in follow-up work. In addition, BPM generates digital signals that can be processed with Deep Learning analysis methods to obtain single-molecule resolution, which gives deep insights into the underlying biomolecular processes.

Compared to sensing with tethered particles^25–28^ (t-BPM), the f-BPM sensing methodology based on free particle motion does not require a tethering molecule, which simplifies sensor preparation, makes the fabrication easier to control, and facilitates preparation for dry storage. In addition, this BPM sensor is insensitive to potential degradation or loss of tether molecules. The free particle motion gives a high contrast between unbound and bound particle states, allowing for accurate detection of state transitions, including between single and multivalent bound states. Furthermore, the freedom of motion enables probing of the complete particle and sensor surface, which opens avenues for achieving a high precision of analyte concentration readings^29,42^.

To conclude, we demonstrated a versatile sensing concept that enables continuous monitoring of macromolecules and small molecules, that permits detection in filtered plasma, can provide single-molecule information, and can operate over many hours without consuming or producing any reagents. In addition, the sensor can be dried, facilitating long-term storage for weeks while maintaining its sensitivity. In the future, the sensor will be combined with a sampling device such as a catheter, the optical readout system will be miniaturized, and the data processing will be further automated. We foresee that this will enable the development of continuous monitoring sensors for applications in fundamental research, for studies on organs on a chip, for the monitoring of patients in critical care, and for the monitoring of industrial processes and bioreactors as well as ecological systems.

## Methods

### Materials

Glass slides (25 × 75 mm, thickness #5, Menzel-Gläser) were obtained from VWR. Custom-made fluid cell stickers with an approximate internal volume of 20 μl were obtained from Grace Biolabs (USA). PBS tablets, NaCl, Cortisol 3-CMO, NHS, DCC-Urea, EDC, HOBt, DIPEA, sucrose, trehalose, and bovine serum albumin (BSA) were purchased from Sigma-Aldrich. NeutrAvidin protein, EZ-Link™ NHS-PEG4-Biotin, Dynabeads MyOne Streptavidin C1 and Dynabeads M-270 Streptavidin were purchased from ThermoFisher Scientific. mPEG-biotin (MW 1kDa) was purchased from Nanocs. Poly(l-lysine)-grafted poly(ethylene glycol) was purchased from SuSoS (Switzerland) with a grafting ratio of 3.5. The molecular weight of the PLL backbone and PEG side chains are 20 and 2 kDa respectively. Azide functionalized PLL-g-PEG (Nanosoft Biotechnology LLC, USA) is composed of a 15 kDa PLL backbone and 2 kDa PEG chain with a grafting ratio of 3.5. The ssDNA oligonucleotides (standard desalting and HPLC purification for chemically modified DNA) were purchased from IDT (Integrated DNA Technologies). The sequence and modification of all ssDNAs are listed in Supplementary Table 3.

### DNA sandwich assay

#### Preparation of biofunctionalized surface

Glass slides (25 × 75 mm, thickness #5, Menzel-Gläser) were cleaned by 30 min of sonication in isopropanol (VWR, absolute) and 30 min of sonication in MilliQ. Afterwards the glass substrate was dried with a nitrogen stream and custom-made fluid cell stickers (Grace Biolabs) with an approximate volume of 20 μL were attached to the substrate. The substrate was washed with 100 μL PBS (130 mM NaCl, 7 mM Na2HPO4, 3 mM NaH2PO4 at pH 7.4). NeutrAvidin protein (ThermoFisher Scientific, 31000) was diluted to 100  μg/mL in PBS and 50 μL was added to the fluid cell, followed by 1 hour of incubation at room temperature (RT).

The substrate-side binder is a partially double-stranded DNA consisting of two complementary oligos, namely a 31 nt oligo (5′ GCA GTC ACG TTC TCG AAT CGA ACA TTA TTA C 3′) and a biotin functionalized oligo (5′ CGA TTC GAG AAC GTG ACT GCT TTT T 3′ Biotin). The 100 μM 31 nt oligo was pre-hybridized with 100 μM biotin functionalized oligo using a thermal cycler and a volume ratio of four to one. The partially double-stranded DNA was diluted to 50 nM in PBS, with an additional 450 nM of 16-nt biotin-polyT (5′ TTT TTT TTT TTT TTT T 3′ Biotin) present for a total DNA concentration of 500  nM. 75 μL of the DNA solution was added to the fluid cell and incubated for 45 min at RT. Subsequently, remaining free NeutrAvidin binding sites were blocked by adding 75 μL of 100 μM 1 kDa mPEG-biotin (Nanocs, PG1-BN-1k) in PBS and incubating for 15 min at RT. As a last blocking step, 75  μL of 1% BSA (Sigma-Aldrich, A3733) in PBS was added to the fluid cell and incubated for 1 h at RT.

#### Preparation of particles

Two microlitres of Dynabeads M-270 Streptavidin (2.8 μm diameter; Invitrogen, 65305) was mixed with 2 μL of 10 μM biotinylated particle-side binder (5′ TCA CGG TAC GA 3′ Biotin). The mixture was incubated for 1 h at room temperature (RT) on a rotating fin (VWR, The Netherlands). Subsequently, 20 μL of 100 μM 1 kDa mPEG-biotin in PBS was added and the mixture was incubated for 15 min at RT on the rotating fin. The particle mixture was washed three times with 200 μL PBS using magnetic separation, followed by the addition of 200 μL 1% BSA in PBS and incubation for 1 h at RT on the rotating fin. The particle solution was sonicated with 10 pulses at 70% amplitude with 0.5 duty cycle (Hielscher UIS250V, Ultrasound Technology), and diluted 1.5 times in PBS.

#### Dose-response experiments for the ssDNA sandwich assay

The fluid cell was washed with 75 μL PBS, after which 75 μL of the diluted particle solution was added, and particles were left to sediment towards the substrate for 10 min before starting measurements. Several 5-min blank measurements were performed after the addition of 75 μL PBS to the fluid cell. Dose-response was measured after 1–2 min injection of 75 μL of various concentrations of the 22-nt ssDNA target (5′ TCG TAC CGT GAG TAA TAA TGC G 3′) or the 22-nt ssDNA with 3-nt mismatch target (5′ TCG TAC CGT GAG TAA ATT TGC G 3′) in PBS. Particle motion was recorded for 5 min after each sample injection.

### DNA competition assay

#### Preparation of biofunctionalized surfaces

Glass slides (25 × 75 mm, #5, Menzel-Gläser) were pre-cleaned by 30 min of sonication in isopropanol (VWR, absolute) and 30 min of sonication in MilliQ. Afterwards the substrate was dried with a nitrogen stream, and 1 min of oxygen plasma was applied to the slides to plasma-oxidize the surface. PLL-g-PEG (SuSoS, PLL(20)-g[3.5]- PEG(2)) and PLL-g-PEG-N_3_ (Nanosoft Biotechnology LLC, USA) were pre-mixed in MilliQ with the ratio of 9:1 by weight at a final concentration of 0.45 and 0.05 mg/mL respectively. Custom-made fluid cell stickers (Grace Biolabs) with an approximate volume of 20 μL were then attached to the substrate and the PLL-g-PEG/PLL-g-PEG-N_3_ polymer mixture was immediately injected to the flow chamber and incubated for three hours. After the polymer self-assembled onto the negatively charged substrate, the unbound or loosely bound polymers were removed by aspirating the solution out of the chamber.

The substrate-side binder is a partially double-stranded DNA consisting of two complementary oligos, namely a 31nt oligo (5′ GCA GTC ACG TTC TCG AAT CGA ACA TTA TTA C 3′) and a DBCO functionalized oligo (5′ CGA TTC GAG AAC GTG ACT GCT TTT T 3′ DBCO). The 100 μM 31 nt oligo was pre-hybridized with 100 μM DBCO functionalized oligo at a volume ratio of four to one. After removing the unbound or loosely bound PLL-g-PEG and PLL-g-PEG-N_3_, the partially double-stranded DNA was diluted to 1 μM with 500 mM NaCl in PBS (130 mM NaCl, 7 mM Na_2_HPO_4_, 3 mM NaH_2_PO_4_ at pH 7.4) and injected into the flow cell, followed by at least 72 h of incubation at room temperature.

#### Preparation of particles

One microlitre of Dynabeads MyOne Streptavidin C1 (1 μm diameter, Invitrogen, 65001) was mixed with 1 μL of 10 μM biotinylated ssDNA linker (5′ TAG TCA GGT TGG ATG TCT AC 3′ Biotin) and 4 μL PBS. The mixture was incubated for 1 h at room temperature (RT) on a rotating fin (VWR, The Netherlands). Subsequently, 1 μL of 100 μM biotin-polyT (5′ TTT TTT TTT TTT TTT T 3′ Biotin) was added and the mixture was incubated for 15 min at RT on the rotating fin. The particle mixture was washed with 0.05 vol.-% Tween-20 (Sigma-Aldrich, P1379) in PBS and reconstituted in 4000 μL of PBS. Right before injecting particles into flow cells, the particle solution was sonicated with 10 pulses at 70% amplitude with 0.5 duty cycle (Hielscher UIS250V, Ultrasound Technology).

#### Dose-response experiments for the ssDNA competition assay

The flow cell was washed with 40 μL of PBS, followed by injecting 40 μL of the particle solution. After 15 min of incubation, the particle-side binders (5′ GTA GAC ATC CAA CCT GAC TAC GTG AGT AAT AAT GCG 3′) were added to the flow cell. The particle-side binder has 20-bp complementarity to the 20-nt ssDNA on the particle and 11-bp complementarity to the substrate-side binder on the surface. When the bound fraction of the f-BPM sensor reached the baseline signal, the unbound particle-side binder was removed and 40 μL of 15-nt ssDNA target (5′ AAA AGC ATT ATT ACT 3′) in PBS was injected into the flow cell within 1–2 min, after which the particle motion was recorded for 5 min.

#### Preparation of a ready-to-use cartridge

The flow cell with the biofunctionalized surface was washed with 20 μL of PBS, followed by the injection of 20 μL sugar mixture (25% w/w sucrose (Sigma-Aldrich, 84097), 10 mM trehalose (Sigma-Aldrich, T9449), 0.05 vol.-%Tween20). After 5 min of incubation, the top layer of the flow cell sticker was carefully removed. The surface was dried for 2 h in a vacuum chamber at room temperature.

One microlitre of Dynabeads MyOne Streptavidin C1 (1 μm diameter; Invitrogen) was mixed with 1 μL of 10 μM biotinylated ssDNA linker (5′ TAG TCA GGT TGG ATG TCT AC 3′ Biotin) and 4 μL PBS. The mixture was incubated for 1 h at room temperature (RT) on a rotating fin (VWR, The Netherlands). Subsequently, 1 μL of 100 μM biotin-polyT (5′ TTT TTT TTT TTT TTT T 3′ Biotin) was added and the mixture was incubated for 15 min at RT on the rotating fin. The particle mixture was washed with 0.05 vol.-% Tween-20 (Sigma-Aldrich) in PBS and reconstituted in 3000 μL of sugar mixture (25% w/w sucrose, 10 mM trehalose, 0.05 vol.-% Tween20). Right before injecting particles into flow cells, the particle solution was sonicated with 10 pulses at 70% amplitude with 0.5 duty cycle (Hielscher UIS250V, Ultrasound Technology). 40 μL of particles were added to the dried surface, thereafter the surface was dried for 2 days in a vacuum chamber at room temperature.

A custom-made fluid cell sticker (Grace Biolabs) was attached to the dried surface. The fluid cell was washed with 60 μL of PBS. After 15 min of incubation, the particle-side binders (5′ GTA GAC ATC CAA CCT GAC TAC GTG AGT AAT AAT GCG 3′) were added to the flow cell. The particle-side binder has 20-bp complementarity to the 20-nt ssDNA on the particle and 11-bp complementarity to the substrate-side binder on the surface. When the bound fraction of the f-BPM sensor reached the baseline signal, the unbound particle-side binders were removed and 40 μL of 15-nt ssDNA target (5′ AAA AGC ATT ATT ACT 3′) in PBS was injected into the fluid cell within 1–2 min, after which the particle motion was recorded in blocks of 5 min.

### Cortisol competition assay

#### Cortisol analogue synthesis

Cortisol 3-CMO was functionalized with NHS and then conjugated to ssDNA-amine following the method described in Yan et al.^26^. Briefly, cortisol−DNA conjugates were obtained by mixing 45 μL of 60 mM Cortisol 3-CMO-NHS ester (Sigma-Aldrich, H6635) with 4 μL of 60 mM HOBt (Sigma-Aldrich; 54802), 4 μL of 300 mM EDC (Sigma-Aldrich; E6383), and 4 μL of DIPEA (Sigma-Aldrich; 387649) in dimethylsulfoxide (DMSO). The reaction mixture was incubated at room temperature for 15 min.

Amine-modified DNA (Amine 5′ TGG TCT TAC CCC TGC CGC AC 3) was diluted to 10 μM in MOPS buffer (50 mM MOPS (Sigma-Aldrich; M1254) and 0.5 M NaCl, pH 8.0), of which 72 μL was added to the mixture and left to react for 16  h (room temperature, 850 rpm). A fresh reaction mixture of cortisol, HOBt, EDC, and DIPEA was prepared as before, incubated for 15 min, added to the amine−DNA mixture and left to react for 6 h. The reaction was quenched by adding 25 μL of 500 mM NH_4_OAc (Sigma-Aldrich; A1542).

The reaction mixture containing cortisol−DNA was dissolved in 0.15 mM NaCl in 98% ethanol, stored at −20 °C for 16 h, followed by spinning down at 17,000 × *g* for 15 min at 4 °C. The pellet was washed a second time (0.15 mM NaCl in 98% ethanol), incubated at −20 °C for 75 min, centrifuged, and washed with 70% ethanol. After incubation at −20 °C for 75  min, it was centrifuged, and the cortisol−DNA was obtained after lyophilization. The cortisol−DNA was dissolved to 25 μM and the conjugation verified using gel electrophoresis with a 15% Urea gel at 150 V for 90 min.

#### Biotinylation C53 antibody

C53 cortisol antibody (ThermoFisher Scientific, MA1-83090) was conjugated using EZ-Link^TM^ NHS-PEG4-biotin (ThermoFisher Scientific, A39259) according to the supplier’s protocol.

#### Preparation of biofunctionalized surfaces

Same as described for the DNA competition assay, but here the substrate-side binder was single-stranded DNA consisting of a DBCO functionalized oligo (DBCO 5′ GTG CGG CAG GGG TAA GAC CA 3′), to which a second strand was hybridized in a later step.

#### Preparation of particles

Two microlitres of Dynabeads MyOne Streptavidin C1 (1 μm diameter; Invitrogen) was mixed with 2 μL of 125 nM biotinylated C53 cortisol antibody. The mixture was incubated for 30 minutes at room temperature (RT) on a rotating fin (VWR, The Netherlands). Subsequently, 5 μL of 5 μM biotin-polyT (5′ TTT TTT TTT TTT TTT T 3′ Biotin) was added and the mixture was incubated for 30 min at RT on the rotating fin. The particle mixture was washed with 500 μl of 0.05 vol.-% Tween-20 (Sigma-Aldrich) in PBS and reconstituted in 300 μL of 0.5 M NaCl in PBS, followed by 30 times dilution. Right before injecting particles into flow cells, the particle solution was sonicated with 10 pulses at 70% amplitude with 0.5 duty cycle (Hielscher UIS250V, Ultrasound Technology).

#### Dose-response experiments for the cortisol competition assay

The fluid cell was washed with 80 μL of 0.5 M NaCl in PBS, followed by injecting 40 μL of the particle solution. After 15 min of incubation, 80 μl of 0.05 vol.-% Tween-20 (Sigma-Aldrich) in PBS was added and incubated for another 15 min twice. Next, 80 μl of 500 pM of ssDNA-cortisol (with 20 bp complementary to the substrate-side binder) in PBST was added and incubated for 30 min, followed by 80 μl of 3 nM of ssDNA-cortisol which was incubated for 20 min to reach a bound fraction of ~0.4. Excess ssDNA-cortisol was removed by washing with 80 μl PBST, and particle motion was recorded in 1–3 blocks of 5 min after each 80 μl sample was injected within 1–2 min (cortisol spiked in PBST).

In the case of the lower analogue concentration, 80 μl of 1 nM of ssDNA-cortisol in PBST was added, incubated for 30  min, followed by another addition of 80 μl of 1 nM analogue, incubated for 20 min. Excess ssDNA-cortisol was removed by washing with 40 μl PBST, and particle motion was recorded for 10 min for each 80 μl injected sample (cortisol spiked in PBST).

### Measurements

Measurements of 2.8 μm diameter particles were performed with a widefield upright microscope (Leica DM6000 B) using brightfield illumination and a high-speed FLIR camera (Sony Grasshopper 3; GS3-U3-32S4M-C) with a field of view of 2048 × 1536 pixels (706 μm × 530 μm). One μm diameter particles were measured with a widefield upright microscope (Leica DMI5000 M) using darkfield illumination and a high-speed FLIR camera (Sony Grasshopper 3; GS3-U3-23S6M-C) with a field of view of 1920 × 1200 pixels (1129 μm × 706 μm). Particles in the field of view were tracked in 1–3 blocks of 5 min at a framerate of 60 Hz using dedicated particle tracking software which applies two-dimensional phasor-based localization^43^. The recorded particle trajectories were further analysed using Deep Learning based data analysis to classify unbound and bound states. Details can be found in Supplementary Notes 1 and 2. The dose-response curves of the bound fraction, switching activity and state lifetimes were fitted with a sigmoidal curve to extract the EC50: $y=\,{y}_{{{{{{\rm{min}}}}}}}+\left({y}_{{{{{{\rm{max}}}}}}}-\,{y}_{{{{{{\rm{min}}}}}}}\right)\cdot \frac{{x}^{n}}{EC{50}^{n}+{x}^{n}}.$

### Statistics and reproducibility

No statistical method was used to predetermine sample size. Dilution factors of functionalized particles were chosen such that a few hundred to ~1000 particles were present in the field of view. This sample size is large enough to provide sufficient statistics, as reflected by the ensemble distributions of *D* measured which show bell-shaped curves with a Gaussian-like distribution in the unbound states and the bound states. Particles non-specifically stuck on the substrate, i.e., particles with an average diffusion coefficient below 0.01 μm/s^2^_,_ particles showing unusually high switching activity (>4 times the standard deviation of the mean switching activity), and colliding particles showing (partially) identical trajectories were excluded from analysis. These particles were removed to limit the bias in the bound fraction, switching activity, and (un)bound state lifetime parameters. In total, <10% of all tracked particles were excluded from the analysis. The experiments were not randomized, and the Investigators were not blinded to allocation during experiments and outcome assessment.

### Reporting summary

Further information on research design is available in the Nature Research Reporting Summary linked to this article.

## Supplementary information

Supplementary Information

Reporting Summary

## Data Availability

Source data are provided with this paper.

## Source data

Source Data

## Code availability

Deep Learning post-processing code is available via 10.5281/zenodo.6974150. Particle dynamics simulation code is available via 10.5281/zenodo.6998256.
