# Supplementary material for: Continuous biomarker monitoring with single molecule resolution by measuring free particle motion
Source: Nat Commun. 2022 Oct 13;13:6052. doi: 10.1038/s41467-022-33487-3 (PMC9561105; doi:10.1038/s41467-022-33487-3)
Supplement: Supplementary file 1 — Supplementary Information [file 41467_2022_33487_MOESM1_ESM.pdf]

SUPPLEMENTARY INFORMATION

Continuous biomarker monitoring with single molecule resolution by measuring free particle motion

Alissa D. Buskermolen[1,2,5], Yu-Ting Lin[3,4,5], Laura van Smeden[1,2,5], Rik B. van Haaften[3], Junhong Yan[4], Khulan Sergelen[1,2], Arthur M. de Jong[2,3], Menno W. J. Prins[1,2,3,4]*

[1]Department of Biomedical Engineering, Eindhoven University of Technology, the Netherlands.
[2]Institute for Complex Molecular Systems (ICMS), Eindhoven University of Technology, the Netherlands.
[3]Department of Applied Physics, Eindhoven University of Technology, the Netherlands.
[4]Helia Biomonitoring, Eindhoven, the Netherlands.
[5]These authors contributed equally: Alissa D. Buskermolen, Yu-Ting Lin, Laura van Smeden.
*email: m.w.j.prins@tue.nl.

Contents

Supplementary Notes 3

1. Particle dynamics 3
    - 1.1 Analysis of particle diffusion over time 4
    - 1.2 Distribution of diffusion coefficients 5
2. Data Processing 7
    - 2.1 Simulation of particle motion 7
    - 2.2 Deep Learning based data analysis 7
    - 2.3 Thresholding based data analysis 10
    - 2.4. Comparison of Deep Learning and Thresholding analysis results 10
3. Comparison of particles and assay chemistries 12
    - 3.1 Particle properties 12
    - 3.2 Comparison of assay chemistries 13
4. Extended data 16
    - 4.1 Reversibility 16
    - 4.2 Specificity 18
    - 4.3 Tunability 19
    - 4.4 Effect of binder density in cortisol competition assay 20
    - 4.5 Acquisition time 22

Supplementary References 23

Supplementary Notes

1. Particle dynamics

Theoretically, particle motion in f-BPM can be described via the overdamped Langevin equation[1]. This equation describes motion of a particle with radius a embedded in a solvent with dynamic viscosity η in the low Reynolds-number regime. The position $\vec{r}(t)$ of a particle is governed by the differential

$$d\vec{r} = \frac{1}{k_B T}\left(\vec{\vec{D}} \cdot \vec{F} + k_B T\, \vec{\nabla} \cdot \vec{\vec{D}}\right) dt + \vec{\vec{H}} \cdot d\vec{w} \tag{1}$$

in which $d\vec{r}$ denotes displacement of a particle over the time step dt. T and k_B are the temperature and Boltzmann constant, respectively. The diffusion tensor $\vec{\vec{D}} = k_B T \vec{\vec{\mu}}$ relates the force $\vec{F}$ working on the particle to its translational velocity $\vec{u}$. The term $\vec{\vec{H}} \cdot d\vec{w}$ is a random fluctuating term. Collisions between the solvent and the colloid cause random motion and are included in $\vec{\vec{H}} \cdot d\vec{w}$. Here, $d\vec{w}$ are increments governed by a Wiener process, while $\vec{\vec{H}}$ is defined as the relation $\vec{\vec{H}} \cdot \vec{\vec{H}}^T = 2\,\vec{\vec{D}}$.

A particle with radius a at a distance h from the substrate is considered as illustrated in panel A of Supplementary Fig. 1. The hydrodynamic interaction between the particle and the substrate causes the particle's diffusivity to decrease while moving towards the substrate. This interaction is included in the diffusion tensor $\vec{\vec{D}}$. A distinction can be made between the diffusivity in the parallel ($D_\parallel$) and perpendicular ($D_\perp$) direction with respect to the substrate by using the mobility matrix $\vec{\vec{\mu}} = \begin{pmatrix} \mu_\parallel & 0 & 0 \\ 0 & \mu_\parallel & 0 \\ 0 & 0 & \mu_\perp \end{pmatrix}$.[2] The coefficients $D_\parallel$ and $D_\perp$ are plotted versus the height h of the particle in Supplementary Fig. 1b. In this graph, $D_\parallel$ and $D_\perp$ are scaled with $D_0 = \frac{k_B T}{6\pi\eta a}$ and h is scaled with a. The coefficients are computed using derivations that assume no-slip boundary conditions at all surfaces[3,4]. In the BPM system, motion parallel to the substrate is measured. Hence, measured motion in BPM is governed by $D_\parallel = k_B T \mu_\parallel$.

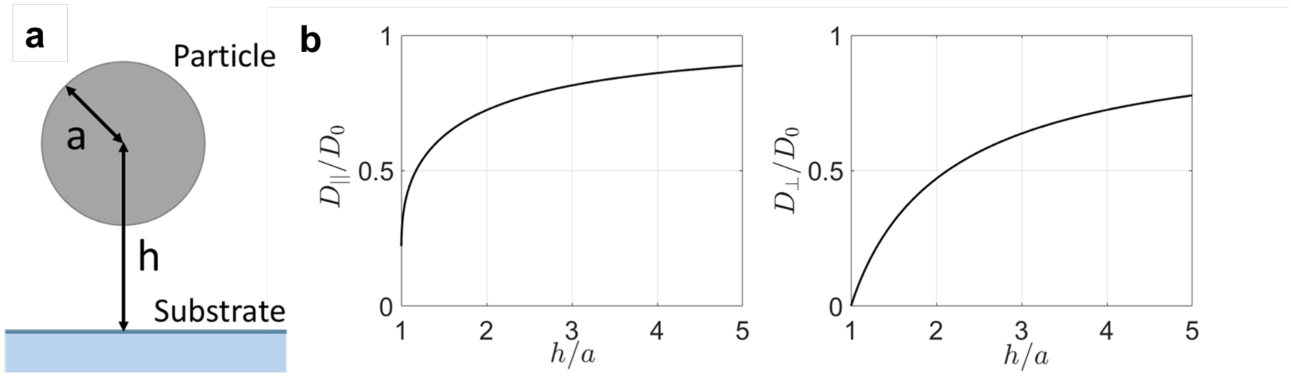

Supplementary Fig. 1: The hydrodynamic interaction between particle and substrate is illustrated. **a**, A particle is considered with radius a. The variable h is defined as the distance between the center of the particle and the substrate. **b**, The diffusion coefficient governing motion parallel ($D_\parallel$; left graph) and perpendicular ($D_\perp$; right graph) is plotted versus the distance h. The distance h is scaled with the particle radius a, and the diffusion coefficients are scaled with $D_0 = \frac{k_B T}{6\pi\eta a}$.

The force $\vec{F}$ working on a particle in the unbound state is governed by gravity

$$\vec{F} = -\frac{4}{3}\pi a^3(\rho_{part} - \rho_{med})g\vec{e}_z = -\frac{4}{3}\pi a^3 \Delta\rho\ g\ \vec{e}_z \tag{2}$$

Here, ρ_{part} is the mass density of the particle, ρ_{med} is the mass density of the solvent, $\Delta\rho$ equals $\rho_{part} - \rho_{med}$, and g is the gravitational acceleration ($g = 9.81\frac{m^2}{s}$). Since only gravity works on the particle, the probability that the center of the particle can be found at a height h is governed by a Boltzmann distribution

$$P(h) = \frac{4\pi a^3 \Delta\rho g}{3k_B T}\exp\left(\frac{-4\pi a^4 \Delta\rho g}{3\ k_b T}\ (h-a)\right), \qquad h \geq a \tag{3}$$

The mean height of the particle center equals $\langle h \rangle = \int_a^\infty h\ P(h)dh = a + \frac{3\ k_B T}{4\ \pi a^3 \Delta\rho g}$. Hence, the average particle-substrate distance equals $\langle h \rangle - a = \frac{3\ k_B T}{4\ \pi a^3 \Delta\rho g}$. At room temperature ($T = 298$ K), the average particle-substrate distance equals $1\ \mu m$ for small particles ($a = 0.5\ \mu m$, $\rho_{part} = 1.8 gr/cm^3$) and 60 nm for large particles ($a = 1.4\ \ \mu m$, $\rho_{part} = 1.6\ gr/cm^3$).

1.1 Analysis of particle diffusion over time

In Supplementary Note 1.2, graphs are shown of the diffusive properties of particles. The calculation of these signals is based on analyzing the mean squared displacement of consecutive intervals of measured particle motion (30 datapoints, framerate of 60 Hz). The measurement window Δt of each interval equals N times dt, in which dt equals the reciprocal of the framerate f of the camera. Then, the particle trajectory $x(t)$ and $y(t)$ within the measurement window Δt can be used to calculate the corresponding mean squared displacement $\langle \Delta r^2(j\ dt)\rangle$ over the timestep $j\ dt$ via[5]

$$\langle \Delta r^2(j\ dt)\rangle = \frac{1}{N - \mathrm{j}}\sum_{i=1}^{N-j}\left[\Big(x\big((i+j)dt\big) - x(idt)\Big)^2 + \Big(y\big((i+j)dt\big) - y(idt)\Big)^2\right] \tag{4}$$

First, $\langle \Delta r^2(j\ dt)\rangle$ is calculated using Supplementary Equation (4) for $j = \{1, n\}$. Then, the corresponding measured diffusion coefficient (here denoted by D_m) is determined via a weighted average

$$D_m = \sum_{j=1}^{n} \omega(j)\frac{\langle \Delta r^2(j\ dt)\rangle}{4\ j\ dt} \tag{5}$$

with $\omega(j)^{-1} = \frac{j(2j^2+1)}{N-j+1}$. This weight factor is equal to the relative variance of $\langle \Delta r^2(j\ dt)\rangle$.[5]

When measuring free motion, the mean squared displacement $\langle \Delta r^2(dt)\rangle$ is linear in time dt via

$$\langle \Delta r^2(dt)\rangle = 4\ D\ dt \tag{6}$$

in which D equals the (parallel) diffusion coefficient and the pre-factor 4 originates from the dimensionality of the measurement. Free diffusion is observed when no bonds are formed between the particle and substrate. In this case, estimates for the particle's parallel diffusivity are determined from the mean squared displacement using Supplementary Equation (4) and Supplementary Equation (5). When a bond is formed between the particle and substrate, confined particle motion is observed. Now, the slope of $\langle \Delta r^2(dt)\rangle$ versus dt is decreased compared to Supplementary Equation (6) and $\langle \Delta r^2(dt)\rangle$ eventually converges to a constant

value for large dt. Meaning that a lower diffusivity signal is observed compared to free diffusion.

1.2 Distribution of diffusion coefficients

The particle-substrate distance varies in time resulting in fluctuation of the particle diffusivity. Distributions of the local diffusivity experienced by a particle are measured via Brownian Dynamics simulations. The found distributions can then be compared to the experimentally obtained distributions in D that correspond to the unbound state in Supplementary Fig. 2. Supplementary Equation (1) is used to model particle motion by numerically implementing a Fixman midpoint scheme[6] in a developed MATLAB code. Furthermore, $\vec{\vec{D}}$ is computed during simulation using derived expressions[3,4], of which the coefficients are shown in Supplementary Fig. 1. A reflective boundary condition is put at $z = 0$ to keep the particle from crossing the substrate.

Simulations are performed for particles that hover freely over a substrate. Distributions of the parallel diffusivity $D_{\parallel}$ are measured from the modeling. Aqueous conditions were assumed ($\eta = 8.9 \cdot 10^{-4}\ Pas$, $T = 298\ K$, $\rho_{med} = 1\ gr/cm^3$) and simulations are performed for the two particle sizes. In both simulations, an integration timestep is used equal to $10^{-5}\tau$ in which $\tau = \frac{a^2}{D_0}$. 200.000 seconds of motion is simulated in each run while the particle position ($\vec{r}$) and parallel diffusivity ($D_{\parallel}$) were stored 60 times per second. Finally, it is shown that the camera's exposure time can affect the measured mean squared displacement[7]. Since the measured diffusivity is based on analyzing the mean squared displacement (Supplementary Equation (5) and Supplementary Equation (6)), motion blur was included in the simulation by averaging the x and y position of the particle during an exposure time of 5 ms for each position acquisition.

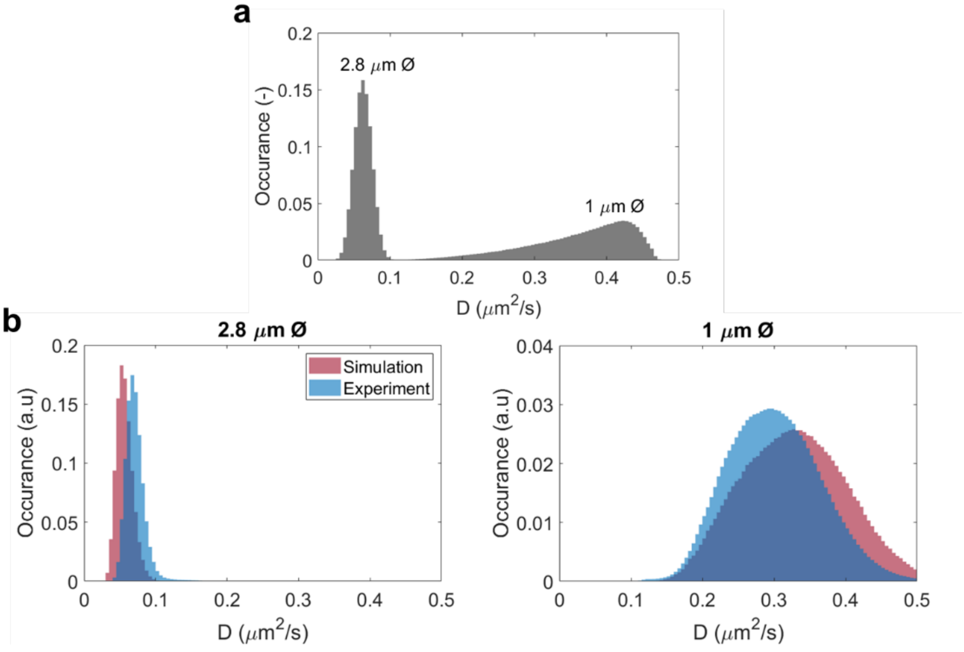

Supplementary Fig. 2: Distributions of the diffusion coefficient (*D*) of simulated and experimentally obtained particle motion. Graphs are shown for particles with a diameter of 1 and 2.8 μm. **a**, Distributions of the experienced parallel diffusivity of simulated particle motion. **b**, Distributions of the measured diffusivity (including motion blur) from simulated particle motion (red) or experimentally obtained particle motion (blue).

The diffusivity (in grey) is shown in Supplementary Fig. 2a for the simulated 1 μm and 2.8 μm particles. The diffusivity of the 1 μm particles varies more than that of the 2.8 μm particles, since a smaller particle can cover a larger range of distances from the substrate. Since a 2.8 μm particle remains close to the substrate, its diffusivity does not vary significantly.

Supplementary Fig. 2b compares the distributions of the diffusivity from simulated particle motion (red graph) and experimentally obtained particle motion (blue graph). Although the distributions are similar in shape, their averages are shifted. For the 1 μm particles, the experimentally measured diffusivity is lower than the diffusivity obtained from simulation. This could be caused by interactions between particles, which was not taken into account in the simulations. The diffusivity of particles decreases when these interactions are present in a sample[8].

The graphs also show that the experimentally observed diffusivity of 2.8 μm particles is higher compared to simulated values. In the BPM system, the substrate is covered with biomolecules, which can induce fluid slip. Fluid slip can increase the particle's diffusivity, most visible when the particle remains close to the substrate, as is the case with the 2.8 μm particles[9]. In the simulations, a no-slip condition is assumed on the substrate, which might cause an underestimation of the particle's diffusivity compared to the experimental set-up.

2. Data Processing

To analyze the BPM data, two different analysis methods were used. The first is a Deep Learning based method, with Deep Learning models that are trained using simulated particle traces. The second is a thresholding method, where the distinction between unbound and bound states is made based on a single threshold at a certain diffusion coefficient value. In this section, we first describe how particle motion was simulated, to serve as input for the training of the deep learning models. Next, the principles of the deep learning data analysis are described, after which a comparison is made with the thresholding method. The distinction of mono- and multivalent bonds is discussed in the section thereafter, and lastly we describe the analysis of state lifetimes.

2.1 Simulation of particle motion

Simulations are performed to generate particle motion as measured in the BPM system by modeling the overdamped Langevin equation (Supplementary Equation (1)) while also including rotational diffusion and torques. The particle is modeled as a sphere with on average 200 spherical protrusions (radius varies between 10 and 60 nm), representing roughness on the particle surface as observed in SEM images. These protrusions are not allowed to overlap with the substrate.

During a simulation run, bonds can form and dissociate between the particle surface and points distributed in a square lattice on the substrate. Up to two bonds can be formed between the particle surface and substrate. When a bond is present, the bond force is simplified by a harmonic oscillator

$$F = -k\,\vec{x} \qquad (7)$$

where k equals the bond stiffness and $\vec{x}$ equals the end-to-end bond distance (~20 nm).

Each state lifetime τ is sampled from an exponential distribution

$$P(\tau) = \kappa\, e^{-\kappa\,\tau} \qquad (8)$$

where the rate constant κ depends on the number of bonds n_b that are present

$$\kappa = \begin{cases} \kappa_{01} & if\ n_b = 0 \\ \kappa_{12} + k_{off} & if\ n_b = 1 \\ 2\,k_{off} & if\ n_b = 2 \end{cases} \qquad (9)$$

Here κ_{01} represents the association rate for a transition from an unbound state to a single bound state, k_{12} the association rate from a single bound state to a double-bound state and k_{off} the dissociation rate of a single bond. After each state lifetime, a bond is formed or dissociated between the particle surface and points on the substrate. At the end of a single-bound state, $P = \frac{\kappa_{12}}{k_{12}+k_{off}}$ equals the probability to go to the double-bound state, while $1 - P$ equals the probability for bond dissociation.

2.2 Deep Learning based data analysis

A deep learning analysis method is developed that can distinguish three mobility states of the particle: unbound, bound, and double bound. The modeled double bound states are considered as multivalent states, since the changes in mobility for particles anchored to the substrate by two bonds or more cannot be distinguished. Deep learning is a subcategory of machine learning, in which a model is trained to recognize patterns within a given input. The used algorithms were developed using Python software, in which the framework from the Keras library was used.

The particle's x,y-trajectory measured during a sliding measurement window is regarded as input, while the corresponding particle state is considered as output. To accurately distinguish single bound and multivalent bound states, two separate models were trained. The first model is trained to detect changes from unbound to any bound state. Motion corresponding to bound states is used as input in the second model, which is trained to detect changes from single bound to multivalent bound states. Both models follow the Neural Network architecture as shown in Supplementary Table 1.

Supplementary Table 1: The Neural Network architecture is shown that is used for event detection in the f-BPM system.

**Layer**
1D CNN (Window 10 frames)
Dropout (50%)
Bidirectional LSTM (40 nodes)
Dropout (50%)
Dense (30 nodes)
Dense (20 nodes)
Dense (1 node)

Supplementary Table 1 shows the Neural Network Architecture consisting of the following layers: i) a one-dimensional Convolutional Neural Network (1D CNN) layer is used with a window size of 10 frames. This layer allows the model to learn patterns that are translation invariant; when a pattern is recognized in a particular domain, it can be recognized anywhere[10]. ii) a Bidirectional Long Short-Term Memory (LSTM) layer consisting of 40 nodes is used. An LSTM layer allows a network to learn long-term dependencies, such that output depends on the order of the input[11]. Bidirectionality means that input is processed in both directions, such that patterns can be recognized which would otherwise be missed[12]. iii) three dense neural network layers are added of 30, 20 and 1 nodes. The final node gives the output of the algorithm.

The neural network is trained using simulated particle motion patterns generated via the method described in Supplementary Note 2.1. The essence of training a network is the minimization of a loss function by altering the network's weight parameters. The loss function is a measure of accuracy of the prediction. In our case, the binary cross-entropy function is used as loss function[13], while the program 'adam' is used during optimization[14]. This program adapts the learning rate during the training process to prevent problems such as finding a local minimum of the loss function (too low learning rate) or the disability to converge (too high learning rate). Finally, dropout layers are added to avoid overfitting[15]. At each training step, the dropout layers randomly deactivate nodes with a frequency of 50% such that the network must build varying representations with the remaining fraction. The accuracy of the model was determined by analyzing simulated datasets (different from training sets) and comparing the true (known) states to the detected states. The accuracy was 99.3±0.2% when distinguishing bound and unbound states, and 98.1±0.2% when distinguishing single and double bound states.

Using the trained models, the Deep Learning analysis can be applied to experimental data. The x,y-trajectories of the tracked particles are used as input. Particles are tracked in real-time using video microscopy and dedicated tracking software. As output the analysis gives several parameters: particle motion patterns and diffusion coefficient time traces (Supplementary Fig. 3), diffusion coefficient distributions (Supplementary Fig. 4a), bound fractions (Supplementary Fig. 5a,b), switching activities (Supplementary Fig. 5c,d) and state lifetimes (Supplementary Fig. 4b). The bound fraction is defined as the ratio between the

population of bound states to the total number of states over time. The switching activity is defined as the average number of events (binding and unbinding) per particle per measurement time. The activity is typically reported in mHz.

After determining the bound and unbound states and their corresponding lifetimes, the cumulative distribution function (CDF) of these lifetimes is calculated, which can be represented as a survival curve (1-CDF). The CDF is fitted with a single or double exponential function to extract the characteristic bound and unbound state lifetimes (Supplementary Fig. 4b). In this example, the majority of the single bound lifetimes are expected to originate from switching between unbound and single bound states, whereas the double bound lifetimes are dominated by switching between single bound and double bound states.

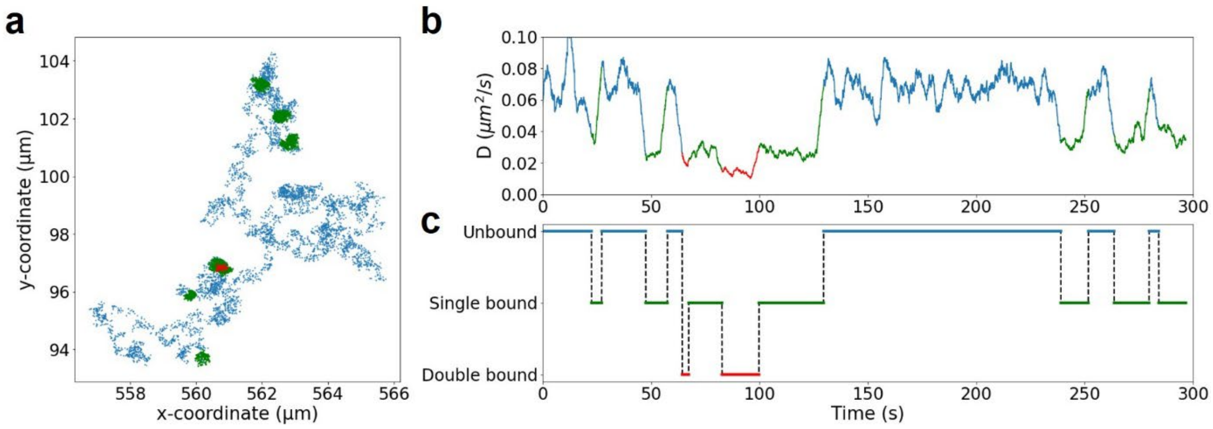

Supplementary Fig. 3: Typical deep learning analysis result. **a**, 2D particle motion pattern with free diffusion (blue), single-molecule binding (green) and multivalent binding (red). **b**, Diffusion coefficient time trace corresponding to the particle motion in panel a. **c**, Detected unbound, single bound and double bound states, corresponding to the time trace in panel b.

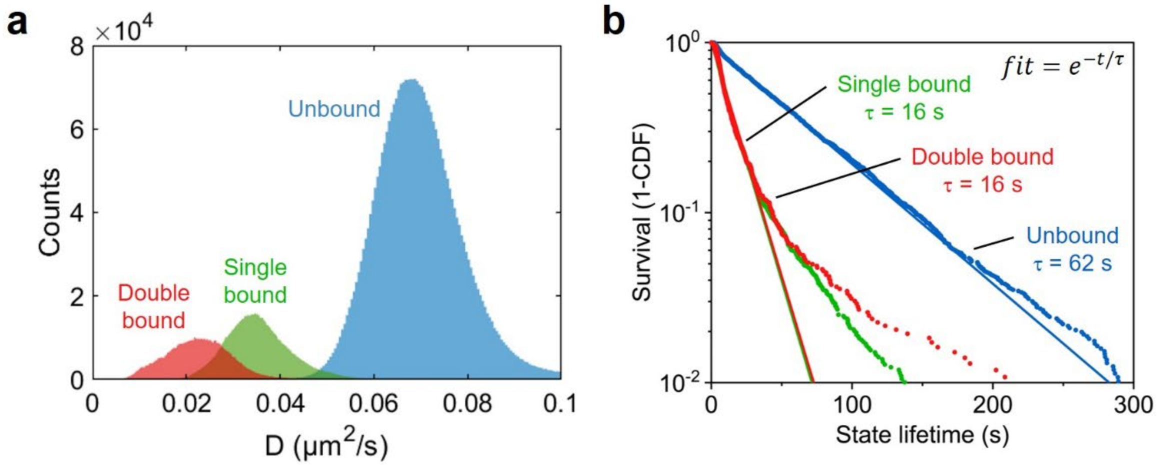

Supplementary Fig. 4: Example of a typical deep learning analysis output for ~500 particles of an oligonucleotide sandwich assay with 120 pM ssDNA target. **a**, The diffusion coefficient distributions show distinct distributions for unbound (blue), single bound (green) and double bound (red) states. The bound fraction is defined as the ratio of single (green) and double bound (red) to the total. **b**, Cumulative distribution functions (CDF) of state lifetimes plotted as survival curves (1-CDF). Distributions of bound states (single bound in green and double bound in red) and unbound states (blue) are fitted with a single exponential curve ($e^{-t/\tau}$) to extract the characteristic bound and unbound state lifetimes t_{SB}, t_{DB} and t_{UB} for a particular analyte concentration.

2.3 Thresholding based data analysis

Thresholding is commonly used in data analysis to signals crossing a certain value to distinguish different populations. A custom Matlab method was used to analyze particle motion and distinguish unbound and bound states based on a single threshold set at a certain value of *D.* Again, the x-y trajectories of the tracked particles are used as input (see Supplementary Note 2.2). The mean squared displacement (see Supplementary Note 1.1) of each particle is calculated over time using a sliding window algorithm. Based on the typical diffusion coefficient distribution of the assay, a fixed *D* threshold is set to distinguish between unbound and bound states. The threshold value depends on the assay studied and is influenced by factors such as particle size and viscosity of the medium. For 1 μm particles in an aqueous solution, a typical threshold for *D* is set between 0.1 and 0.15 $\mu m^2/s$. For 2.8 μm particles this threshold is set lower, because of the slower *D* of larger particles. Typical values lie between 0.04 and 0.05 $\mu m^2/s$. The output parameters are similar to the deep learning data analysis output, except that single and double bound states cannot be distinguished.

2.4. Comparison of Deep Learning and Thresholding analysis results

In Supplementary Fig. 5, dose-response curves of an oligonucleotide sandwich assay analyzed with the Deep Learning method (DL, black data presented in Main Fig. 3c) and with the Thresholding method (TH) are compared. The single-molecule response is shown in red, the multivalent response in blue, and the total response in black. The curves are fitted with a sigmoidal curve to extract the EC50 values (indicated in the graphs), and the shaded area indicates the 95% confidence interval of the fit. For both methods, the bound fraction and the activity parameter show comparable trends, with a characteristic sigmoidal shape of the dose-response.

When comparing the results obtained with both methods, several things stand out. For the bound fraction, the total response (black triangles, Supplementary Fig. 5a,b) is very similar, with EC50 values of 238 ± 40 pM for the DL method, and 248 ± 40 pM for the TH method. However, the DL method gives more information about the type of interactions that are observed at a certain analyte concentration. The single-molecule response (red triangles) starts at low concentrations and reaches a plateau at ~300 pM target. The multivalent response (blue triangles) becomes visible at higher concentrations but does not yet reach a top plateau at 1000 pM target. The total response (black triangles) has a wider dynamic range compared to the two individual contributions (single-molecule and multivalent), illustrating the importance of incorporating both in the analysis.

This also becomes apparent in the activity parameter: the amplitude of the total response observed with the TH method (Supplementary Fig. 5d) is much lower than that of the DL method (Supplementary Fig. 5c). Transitions between single and multivalent bonds are not taken into account in the TH method. As such, the dose-response of the activity from the TH method closely resembles the activity profile for transitions between unbound and single bound states from the DL method. As can be seen in Supplementary Fig. 5c, the single-molecule response (red) starts at low concentrations and reaches a plateau at ~200 pM target, which is a slightly lower concentration compared to the response of the bound fraction (Supplementary Fig. 5a). The same trend is observed using the TH method (Supplementary Fig. 5d). In both cases, the last two data points (open squares) are not included in the fits. These values are significantly lower than the apparent plateau that is reached already. This is likely caused by the increase in multivalent binding as can be seen in Supplementary Fig. 5c (blue squares): transitions between unbound and single bond states occur less and therefore the corresponding activity also decreases at these high concentrations. The multivalent response (blue squares) becomes visible from ~100 pM and starts to reach a top plateau at

1000 pM target. The combined response has a characteristic sigmoidal shape with a clear bottom and top plateau.

All in all, the DL method provides more detailed information about the type of interactions that are observed in the assay compared to the TH method. This is valuable for the characterization of the specific molecular interactions, such as kinetics and state lifetimes. It also allows for better tuning of the assay regarding the single-molecule and multivalent regime.

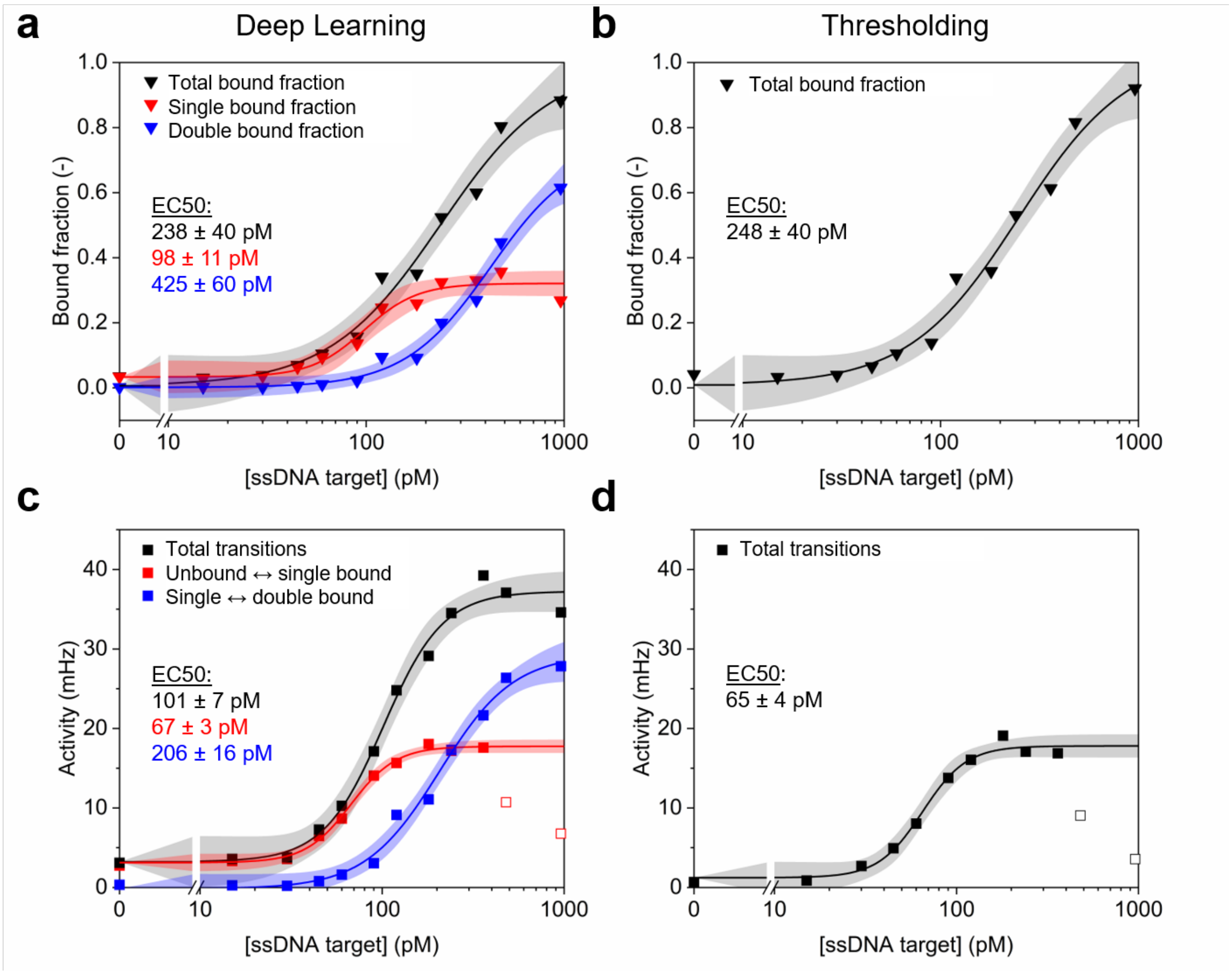

Supplementary Fig. 5: Comparison of dose-response curves of an oligonucleotide sandwich assay analyzed with the Deep Learning method (DL, black data presented in Main Figure 3c) and with the Thresholding method (TH). In red the single-bond response, in blue the multivalent response, and in black the total response. Curves are fitted with a sigmoidal curve and the shaded area indicates the 95% confidence interval of the fit which determines the error in the reported EC50 values. **a**, Dose-response of the bound fraction (mean values) analyzed with the DL method. The EC50 values for the single-bound, multivalent and total response are 98 ± 11, 425 ± 60 and 238 ± 40 pM respectively. **b**, Dose-response of the bound fraction (mean values) analyzed with the TH method. The EC50 is 248 ± 40 pM, which is comparable to the total response in panel **a**. **c**, Dose-response of the activity (mean values) analyzed with the DL method, with EC50 values of 67 ± 3, 206 ± 16 and 101 ± 7 pM for single-bound, multivalent and total interactions respectively. Open squares are not included in the fit. **d**, Dose-response of activity (mean values) analyzed with the TH method, with an EC50 of 65 ± 4 pM, which is comparable to the single-bond response observed in panel **c**. Open squares are not included in the fit.

3. Comparison of particles and assay chemistries

3.1 Particle properties

Supplementary Table 2: List of properties of 1μm and 2.8μm diameter particles.

**Diameter**	**1 μm**	**2.8 μm**
**Product name**	Dynabeads MyOne Streptavidin C1	Dynabeads M-270 Streptavidin
**Material**	Paramagnetic core within a polymer shell; covalently attached recombinant streptavidin	
**Surface type**	Hydrophilic	
**Mass density**	1.8 g/cm^3	1.6 g/cm^3
**Binding capacity per mg beads**	Biotinylated IgG: up to 20 μg<br>Biotinylated ssDNA: ~500 pmol	Biotinylated IgG: up to 10 μg<br>Biotinylated ssDNA: ~200 pmol
**Isoelectric point**	pH 5.2	pH 4.5
**Zeta potential at pH 7**	~35 mV	~50 mV
**Barometric height in water**	~1 μm	~60 nm
**Theoretical D in water ($\mu m^2/s$)**	~0.45	~0.15

We used particles with a diameter of 1 μm and 2.8 μm, fabricated by the same supplier using similar methods. Particles were selected based on earlier experiences with tethered particles[16]: 1 μm particles match the size used in t-BPM, and the barometric height of 2.8 μm particles is comparable with the tether length used in t-BPM experiments. For the competition assays the 1 μm particles were selected because of their high mobility, good contrast between bound and unbound states, and sensitivity in the nanomolar range. For the DNA sandwich assay the 2.8 μm particles were used as these demonstrated higher binding statistics and better sensitivity (picomolar range) compared to the 1 μm particles.

3.2 Comparison of assay chemistries

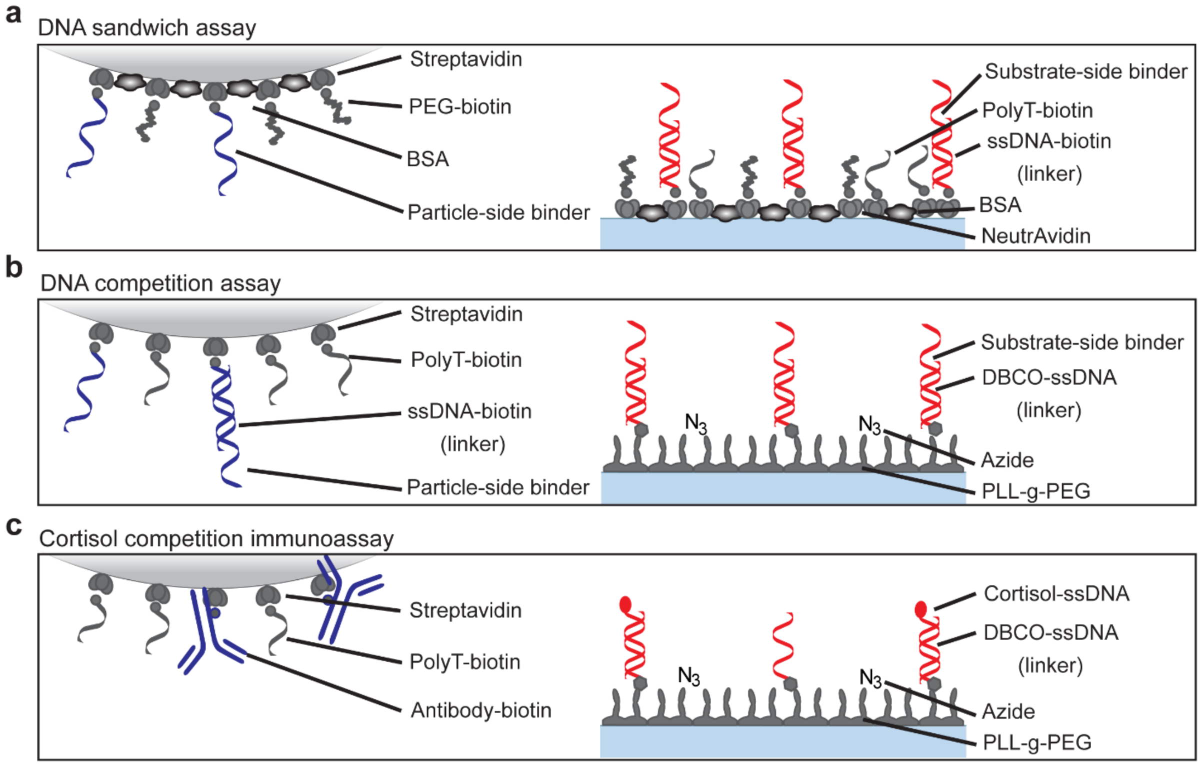

Supplementary Fig. 6: Assay chemistry details. **a**, DNA sandwich assay with 2.8 μm particles. Surface is functionalized with NeutrAvidin and substrate-side binders hybridized to a ssDNA-biotin linker. Free NeutrAvidin binding sites are blocked with 16-nt biotin-polyT and 1K biotin-mPEG. Particles are functionalized with ssDNA-biotin particle-side binders and blocked with mPEG-biotin. Both particles and surface are blocked with BSA. **b**, DNA competition assay with 1 μm particles. Surface is functionalized with PLL-g-PEG, and substrate-side binders pre-hybridized to a DBCO-ssDNA linker are covalently coupled to the PLL-g-PEG layer via the integrated azide groups using click chemistry. Particles are functionalized with ssDNA-biotin linkers and blocked with biotin-polyT. ssDNA particle-side binders are hybridized to the ssDNA linkers. **c**, Cortisol competition assay with 1 μm particles. Surface is functionalized with PLL-g-PEG, and DBCO-ssDNA linkers are covalently coupled to the PLL-g-PEG layer via the integrated azide groups using click chemistry. Cortisol-ssDNA analogues are hybridized to the ssDNA linkers. Particles are functionalized with biotinylated cortisol antibodies and blocked with biotin-polyT.

Supplementary Table 3: Oligonucleotide sequences and modifications. Sequences are numbered in order of appearance in the Methods section.

#	Oligonucleotide name	Sequence and modification
**1**	31nt oligo (hybridized to #2 or #7)	5’ GCA GTC ACG TTC TCG AAT CGA ACA TTA TTA C 3’
**2**	Biotin functionalized oligo (hybridized to #1)	5' CGA TTC GAG AAC GTG ACT GCT TTT T 3' Biotin
**3**	Biotin-polyT	5’ TTT TTT TTT TTT TTT T 3’ Biotin
**4**	Biotinylated particle-side binder (DNA sandwich assay)	5’ TCA CGG TAC GA 3’ Biotin
**5**	22-nt ssDNA target (DNA sandwich assay)	5' TCG TAC CGT GAG TAA TAA TGC G 3'
**6**	22-nt ssDNA with 3-nt mismatch target (DNA sandwich assay)	5' TCG TAC CGT GAG TAA ATT TGC G 3'
**7**	DBCO functionalized oligo (hybridized to #1; DNA competition assay)	5' CGA TTC GAG AAC GTG ACT GCT TTT T 3' DBCO
**8**	Biotinylated ssDNA linker (hybridized to #9; DNA competition assay)	5’ TAG TCA GGT TGG ATG TCT AC 3’ Biotin
**9**	Particle-side binders (hybridized to #8; DNA competition assay)	5’ GTA GAC ATC CAA CCT GAC TAC GTG AGT AAT AAT GCG 3’
**10**	11-nt ssDNA target (DNA competition assay)	5' AAA AGC ATT ATT ACT 3'
**11**	ssDNA-amine (hybridized to #12; Cortisol competition assay)	Amine* 5' TGG TCT TAC CCC TGC CGC AC 3'
**12**	DBCO functionalized oligo (hybridized to #11; Cortisol competition assay)	DBCO 5’ GTG CGG CAG GGG TAA GAC CA 3’

*Amine is used for conjugation to cortisol before hybridization.

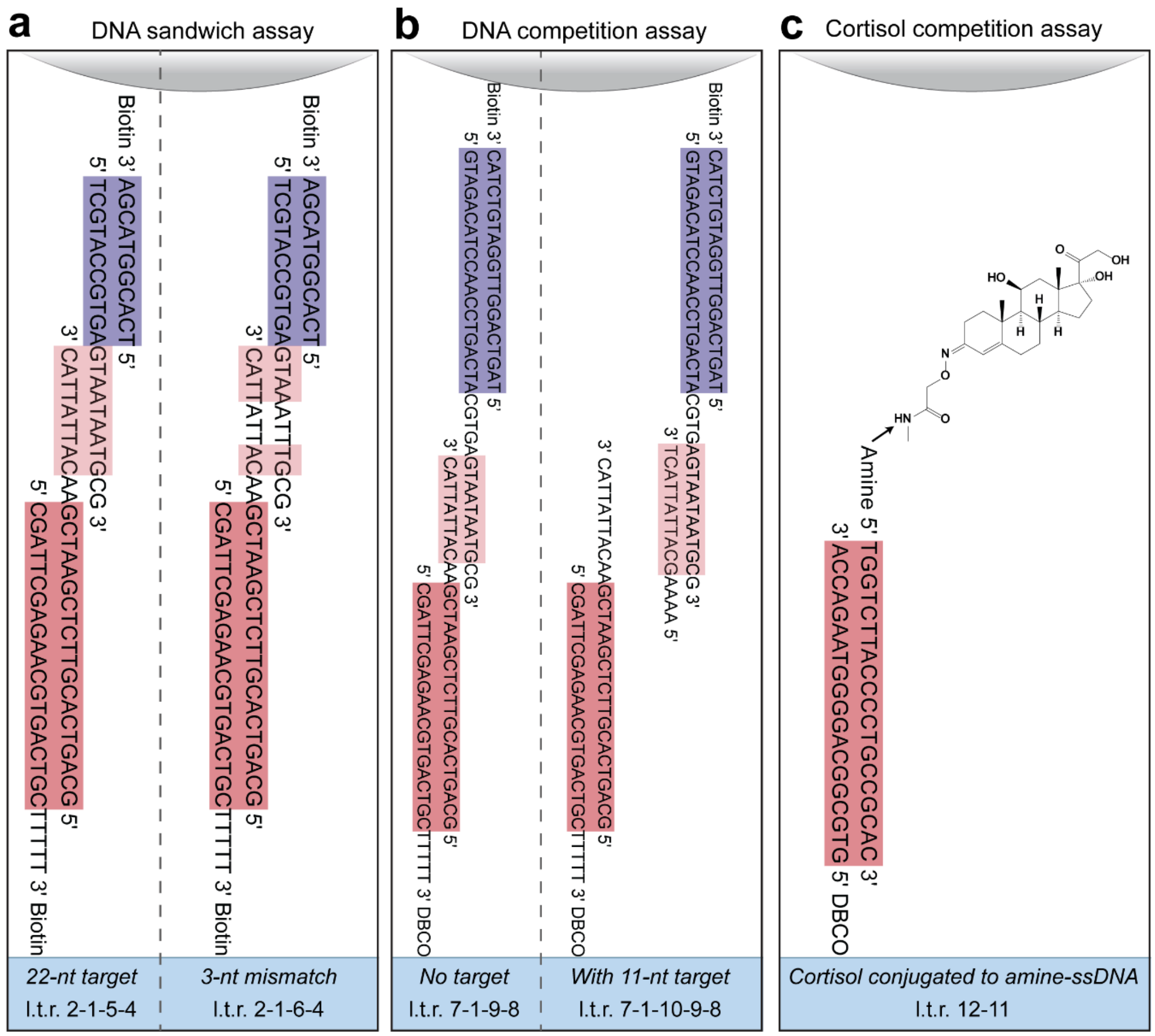

Supplementary Fig. 7: DNA architecture of the assays. **a**, DNA sandwich assay with target (left side; sequence nr. 5 of Supplementary Table 3) and with the 3-nt mismatch (right side; sequence nr. 6). **b**, DNA competition assay without target (left side), and with target (right side; sequence nr. 10). **c**, Part of the cortisol competition assay with cortisol analogue (based on conjugation of cortisol with sequence nr. 11) hybridized to the substrate-side oligo. Particle functionalization is not shown.

4. Extended data

4.1 Reversibility

For continuous monitoring applications, reversibility of the assay is important, because it allows the sensor to function over long timespans while remaining sensitive to varying target concentrations. In this section, additional data is supplied on the reversibility of the oligonucleotide sandwich and competition assays. The reversibility of the cortisol competition immunoassay is discussed in Supplementary Note 4.4.

Oligonucleotide sandwich assay

The reversibility of the response (bound fraction and unbound state lifetime) of the oligonucleotide sandwich assay is demonstrated in Supplementary Fig. 8.

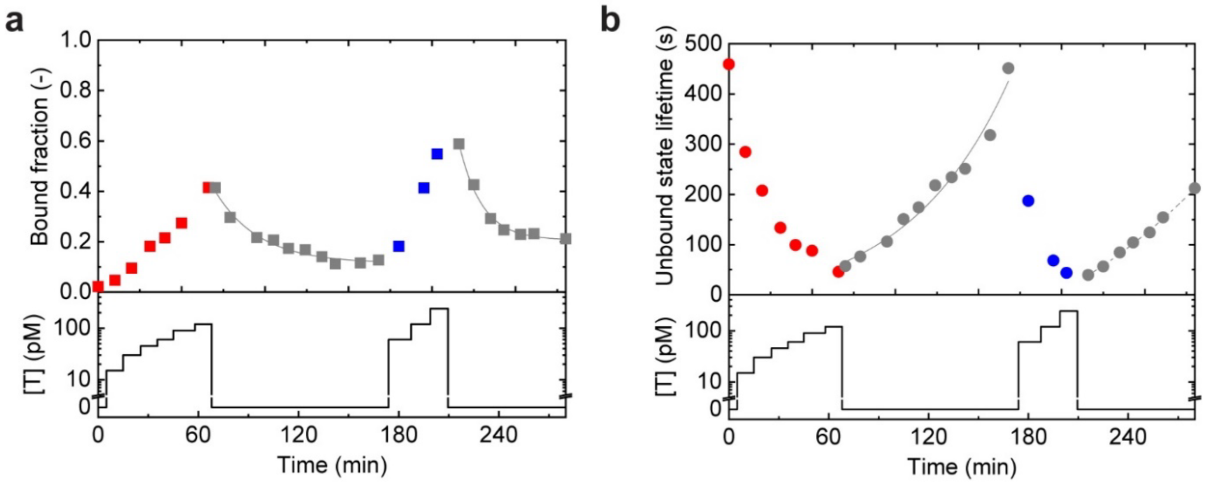

Supplementary Fig. 8: Dynamic response of the oligonucleotide sandwich assay (top panels) to different 20nt target concentrations (bottom panels). **a**, Response expressed as the bound fraction, showing an increasing fraction with increasing target concentration, and reversibility to a base level by removal of target. A second series of increasing target concentrations was applied, and subsequent removal of target demonstrates the stability of the assay over time. Characteristic relaxation times extracted from exponential decay fits (solid grey lines) are 26±4 and 14±2 minutes. **b**, Response expressed as the characteristic unbound state lifetimes (in seconds). A decrease in characteristic unbound state lifetimes is observed upon addition of increasing concentrations of 20nt target, because of an increase in binding probability of particles to the substrate. The system reverses to a base level upon removal of target, with a characteristic relaxation time of 62±17 minutes (single exponential fit, solid grey line). Another sequence of increasing target concentration and removal of target indicates the stability of the system over time.

Oligonucleotide competition assay

The reversibility of the DNA competition assay is demonstrated by recording the switching activity and bound fraction as a function of time. For a competition assay, the switching activity and bound fraction rise with decreasing target concentration. Supplementary Fig. 9 shows the applied concentration profiles of particle binder and 11nt target (bottom panels) and the measured switching activity and bound fraction as a function of time (top panels). The target concentration was first decreased in a step-wise fashion (red squares), and thereafter the target was removed. The competition assay shows a faster response as compared to the sandwich assay. The second dose-response (blue squares) was measured with a similar concentration series. It is expected that the substrate-side binders on the surface should not dissociate over time, causing the system to show a stable baseline activity at zero analyte concentration. As shown in Supplementary Fig. 9, similar levels of bound fraction and switching activity were measured at zero target concentration over a measurement time of approximately 8 hours.

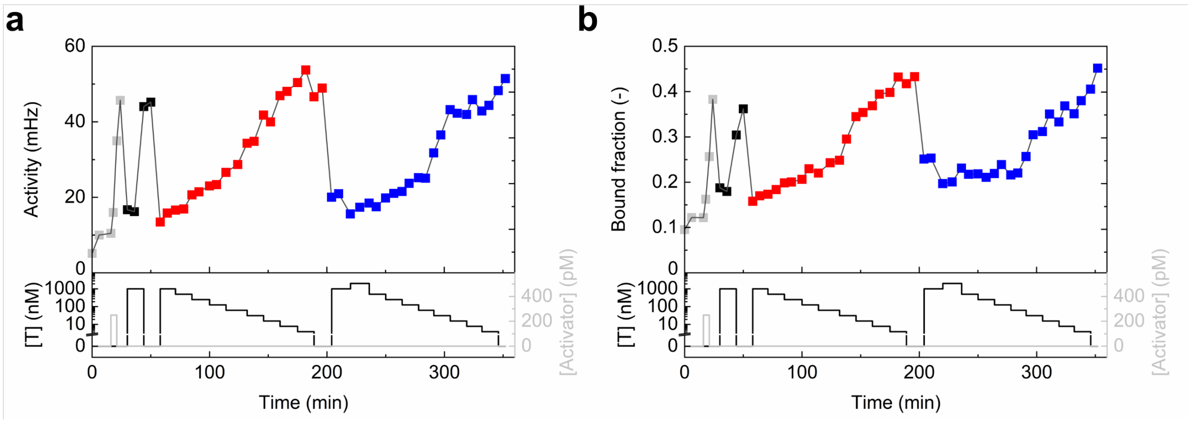

Supplementary Fig. 9: Dynamic response of the DNA competition assay. The red and blue data in the top panels represent two consecutive dose-responses with similar decreasing concentration series applied in a stepwise fashion. The reversibility of the biosensor is demonstrated by recording the switching activity and bound fraction as a function of time before and after the removal of targets. **a**, The top panel shows the switching activity measured over time with the applied concentration profile. The bottom panel shows the applied concentration profiles of particle-side binder (grey) and 11nt target (black). **b**, The dynamic response of the biosensor in terms of bound fraction. The bottom panel shows the applied concentration profiles.

4.2 Specificity

The specificity of the oligonucleotide sandwich assay was tested by introducing a ssDNA target molecule which has a 3-nt mismatch with the binders. In the same sensor cartridge, the response in the presence of buffer for a 120 pM mismatched target or 120 pM target was recorded. Measurements were performed twice, with a measurement in buffer in between. The 120 pM mismatched target samples are indistinguishable from the measurements in buffer, whereas 120 pM target shows a significant increase in both bound fraction and activity (Supplementary Fig. 10).

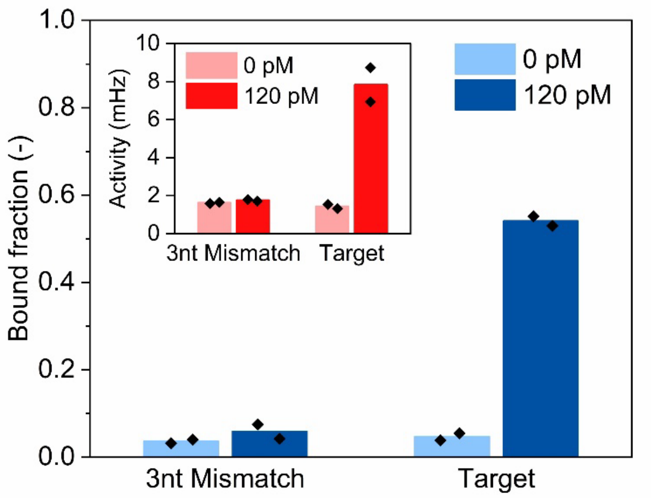

Supplementary Fig. 10: Specificity of the oligonucleotide sandwich assay was tested using a mismatched target molecule which has a 3nt mismatch in the sequence. The response of the assay was measured after addition of either PBS or 120 pM of target or mismatched target. Measurements were repeated twice (black diamonds). No significant difference is observed between the PBS sample and the mismatched target sample, whereas a significant difference is observed between the PBS sample and the 20nt target sample, both in the bound fraction and the activity.

4.3 Tunability

The response range of the oligonucleotide sandwich assay can be tuned in several ways, for example by changing the density of the substrate-side binder (SB09; 9-bp complementarity to ssDNA target). Supplementary Fig. 11 shows the effect of a 10-fold change in binder incubation concentration on the response in terms of activity, bound fraction, and unbound state lifetimes. Increasing the binder concentration from 5 nM to 500 nM shifts the sensitivity of the assay over several orders of magnitude, to the low picomolar range. This indicates that the sensor can easily be adapted to be sensitive in different concentration ranges, without the need to change the binder affinity.

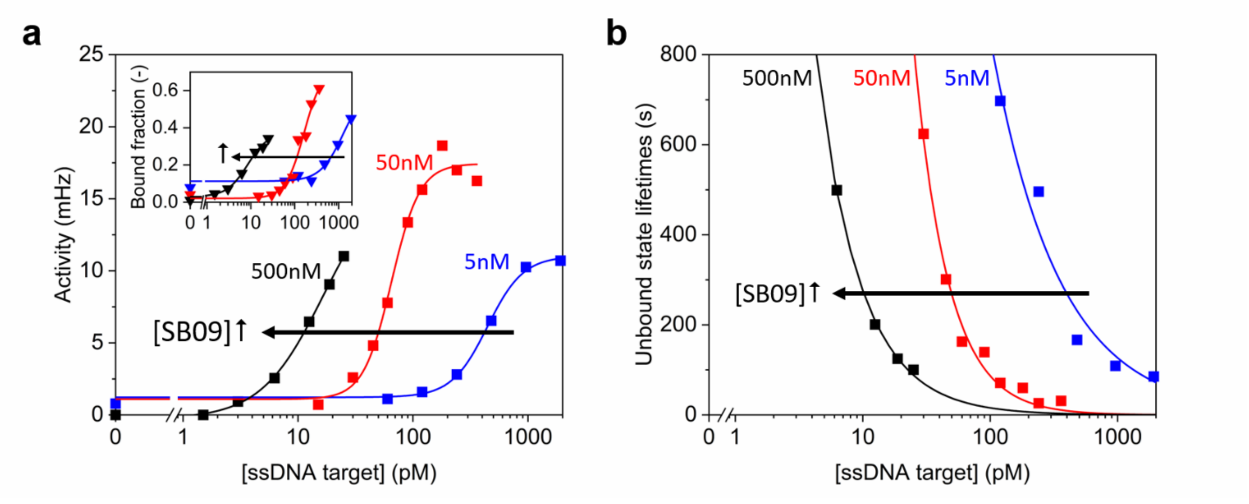

Supplementary Fig. 11: Tunability of an oligonucleotide sandwich assay, by changing the substrate-side binder concentration. **a**, Effect of 10-fold change of SB09 concentration on the activity and bound fraction dose-response. For both parameters, a shift of the curves to lower concentration is observed when the substrate binder concentration is increased from 5 to 50 to 500 nM. A sigmoidal curve was fitted to the data to illustrate the shift in dose-response. **b**, The effect on the unbound state lifetimes dose-response is similar, with higher concentrations of SB09 resulting in a higher sensitivity. Curves were fitted with a power function as a guide to the eye. These results show that by changing a single assay parameter (the substrate binder concentration), the dynamic range of the sensor can easily be shifted over several orders of magnitude.

4.4 Effect of binder density in cortisol competition assay

We investigated how the analogue density on the substrate influences the response of the cortisol sensor (switching activity, bound fraction, state lifetimes, relaxation). The analogue density was varied by changing the analogue concentration during the step where cortisol-ssDNA conjugates hybridize to ssDNA linkers on the PLL-g-PEG coated substrate. In this process step, the linker density on the PLL-g-PEG layer was in excess compared to the analogue density; the linker was supplied at 1 μM during its surface coupling process, while the analogue was supplied at a few nM.

Supplementary Fig. 12 shows experimental results for 3 nM (filled squares) and 1 nM (open squares) analogue concentration supplied, to study the effect of the substrate analogue density on the sensor response. The data show that the switching activity (panels a-c) is approximately a factor two lower in case of the lower analogue density, while the EC50 values are comparable (1.19±0.48 μM and 2.48±1.76 μM). The data on bound fraction (panels d-f) are quite comparable for the two different analogue densities, with EC50 values of 1.49±0.57 μM and 1.13±1.11 μM.

Our understanding of the sensor is that the rate-limiting process is the association between particle and substrate, which is on the one hand determined by the encounter rate of particle and substrate, and on the other hand by the density of available antibodies on the particle and the density of analogues on the substrate. The encounter rate of particle and substrate is governed by physical processes as described in Supplementary Note 1. Since the same 1 μm particles, with the same antibody density, and the same solutions were used, the encounter rate did not change between the different experiments. However, in the case of lower analogue density, it takes a particle with the same particle-to-substrate encounter rate a longer time to reach an analogue molecule and form a bond. This is in agreement with observing a decrease in the switching activity for the lower analogue density condition. However, further research is needed to confirm this.

As mentioned above, the data shows that the fitted EC50 values do not depend on the analogue density. In our understanding of the cortisol BPM sensor, the EC50 relates to the occupation of antibodies on the particles by cortisol from solution. The occupation of antibodies is determined by Langmuir kinetics and not by the density of analogues on the substrate, in agreement with the experimental results in Supplementary Fig. 12.

The dependence of relaxation rate on analogue density is not clear from Supplementary Fig. 12; the activity data suggests a faster relaxation for lower analogue density, but the bound fraction seems to show the opposite. This is a topic for further research.

Panels g-i show the mean bound state lifetimes determined from single-exponential fits of the bound state survival curves. The data are comparable for the two different analogue densities, with quite some scatter on the data, confirming that the bound state lifetime does not depend on the analogue areal density. At low cortisol concentrations, the measured lifetimes are in the range of 5-10 seconds. We attribute these lifetimes to single-molecule interactions between antibody on the particle and cortisol-analogue on the substrate. At high cortisol concentrations (~1 μM and higher), the bound-state lifetimes are lower. The shorter lifetimes are likely the result of the blocking of antibody binding sites by cortisol, causing the bound states to be dominated by short-lived non-specific interactions rather than longer-lived interactions between antibodies and cortisol-analogue.

The experiments reported in the body text (Supplementary Fig. 5) were performed with 3 nM cortisol analogues supplied to the substrate, with the main advantage that it gives a higher switching activity signal.

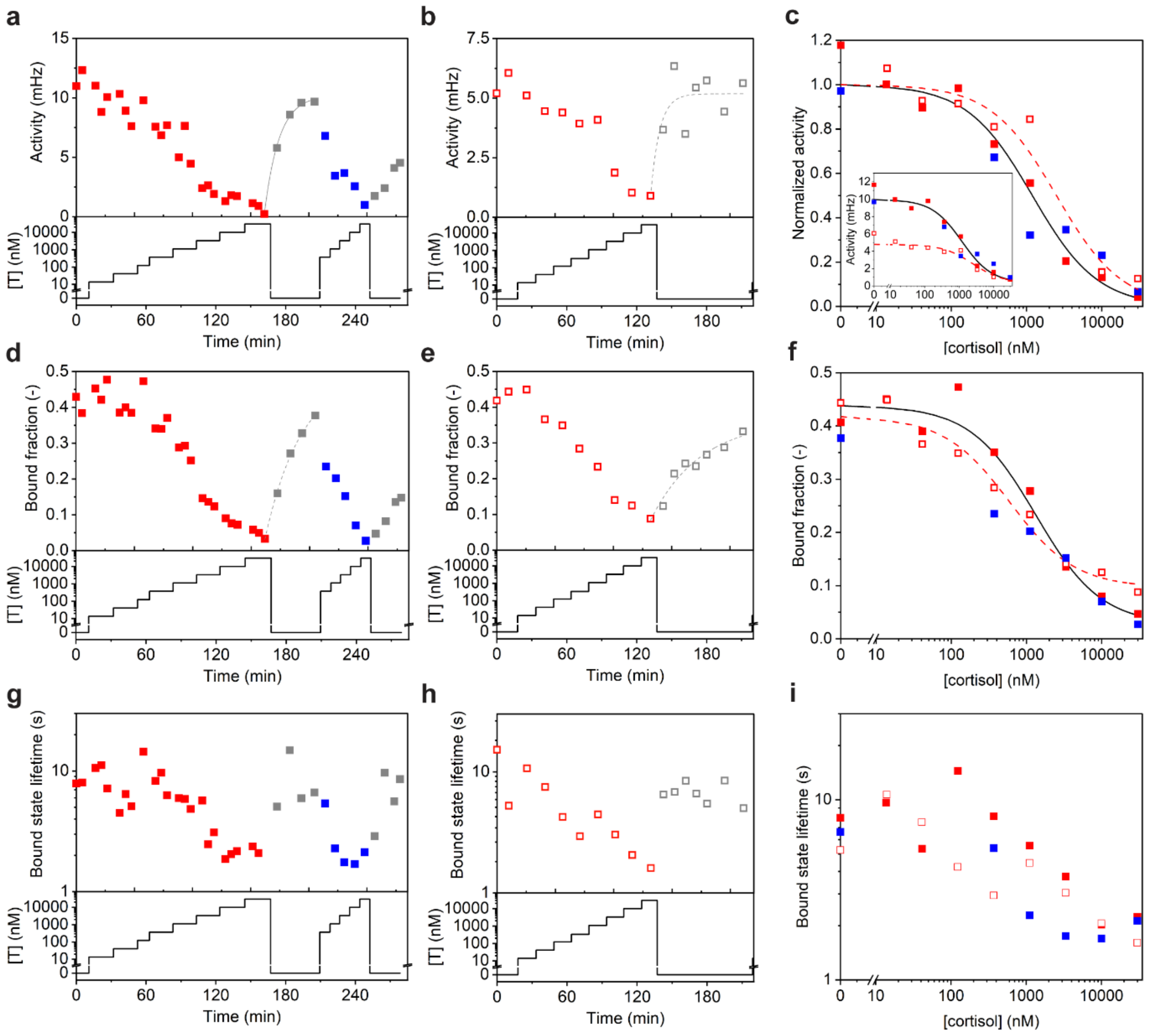

Supplementary Fig. 12: Cortisol competition immunoassay for two analogue densities on the substrate; solid symbols for high density, open symbols for low density. Panels **a-c** show activity data. Panels **d-f** show bound fraction data. Panels **g-i** show bound state lifetime data. Cortisol concentrations were applied over time, as indicated in the graphs. Blank solutions (grey data points) were applied to study the relaxation of the sensor. Panels **c**, **f**, and **i** show the parameters as a function of concentration, derived from the data in the other panels. In panel **c**, sigmoidal curves were fitted, with EC50 values of 1.19±0.48 μM (black) and 2.48±1.76 μM (red dashed line). The inset shows the data before normalization. In panel **f**, sigmoidal curves were fitted, with EC50 values of 1.33±0.24 μM (black) and 1.13±1.11 μM (red dashed line).

4.5 Acquisition time

For the continuous monitoring application, the time required for recording a reliable bound fraction was investigated in a cortisol assay, see Supplementary Fig. 13. The graph shows that the curves are very similar for 2 sec, 1 min, and 10 min recording. This shows that 2 seconds of video recording (framerate of 60 Hz) is sufficient to determine the fraction of bound particles.

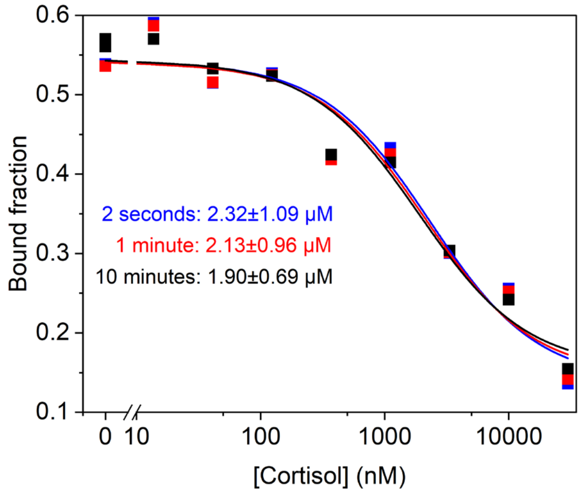

Supplementary Fig. 13: Bound fraction dose-response curve of the cortisol assay (with ~650 particles), recorded using different measurement durations. The data shows that bound fractions can be reliably determined with only 2 seconds observation time.

Supplementary References

1. Reichert, M. Hydrodynamic Interactions in Colloidal and Biological Systems. (University of Konstanz, Konstanz, 2006).
2. Korn, C. B. & Schwarz, U. S. Mean first passage times for bond formation for a Brownian particle in linear shear flow above a wall. *J. Chem. Phys.* **126**, 095103 (2007).
3. Cichocki, B. & Jones, R. B. Image representation of a spherical particle near a hard wall. *Phys. A Stat. Mech. its Appl.* **258**, 273–302 (1998).
4. Perkins, G. S. & Jones, R. B. Hydrodynamic interaction of a spherical particle with a planar boundary. II. Hard wall. *Phys. A Stat. Mech. its Appl.* **189**, 447–477 (1992).
5. Qian, H., Sheetz, M. P. & Elson, E. L. Single particle tracking. Analysis of diffusion and flow in two-dimensional systems. *Biophys. J.* **60**, 910 (1991).
6. Delong, S., Balboa Usabiaga, F. & Donev, A. Brownian dynamics of confined rigid bodies. *J. Chem. Phys.* **143**, 144107 (2015).
7. Mortensen, K. I., Flyvbjerg, H. & Pedersen, J. N. Confined Brownian Motion Tracked With Motion Blur: Estimating Diffusion Coefficient and Size of Confining Space. *Front. Phys.* **8**, (2021).
8. Hsiao, L. C., Saha-Dalal, I., Larson, R. G. & Solomon, M. J. Translational and rotational dynamics in dense suspensions of smooth and rough colloids. *Soft Matter* **13**, 9229–9236 (2017).
9. Lee, T., Charrault, E. & Neto, C. Interfacial slip on rough, patterned and soft surfaces: A review of experiments and simulations. *Adv. Colloid Interface Sci.* **210**, 21–38 (2014).
10. Goodfellow, I., Bengio, Y. & Courville, A. *Deep Learning*. (MIT Press, Cambridge, MA, 2016).
11. Hochreiter, S. & Schmidhuber, J. Long Short-Term Memory. *Neural Comput.* **9**, 1735–1780 (1997).
12. Schuster, M. & Paliwal, K. K. Bidirectional recurrent neural networks. *IEEE Trans. Signal Process.* **45**, 2673–2681 (1997).
13. Hastie, T., Tibshirani, R. & Friedman, J. *The Elements of Statistical Learning*. (Springer, New York, 2009).
14. Kingma, D. P. & Ba, J. L. Adam: A method for stochastic optimization. in *ICLR 2015, 3rd International Conference on Learning Representations* (Ithaca, New York, 2015).
15. Srivastava, N., Hinton, G., Krizhevsky, A., Sutskever, I. & Salakhutdinov, R. Dropout: A simple way to prevent neural networks from overfitting. *J. Mach. Learn. Res.* **15**, 1929–1958 (2014).
16. Visser, E. W. A., Yan, J., Van IJzendoorn, L. J. & Prins, M. W. J. Continuous biomarker monitoring by particle mobility sensing with single molecule resolution. *Nat. Commun.* **9**, (2018).
